# Human iPSC models reveal *APOE4*-induced microglial remodeling and extracellular vesicle dysregulation in AD pathogenesis

**DOI:** 10.21203/rs.3.rs-8594804/v1

**Published:** 2026-09-10

**Authors:** Yang You, Son N. Nguyen, Sean D. Mann, Prakruthi Vadakattu, Takahisa Kanekiyo, Zhengrong Zhang, Nibedita Basu Ray, Angela Duong, Tony Tuck, Julia TCW, Alison M. Goate, Celeste M. Karch, Scott A. Shaffer, Seiko Ikezu, Maria Paz Gonzalez-Perez, Wayne W. Poon, Tsuneya Ikezu

**Affiliations:** 1 Department of Neuroscience, Mayo Clinic, Jacksonville, FL, USA; 2 Mass Spectrometry Facility, University of Massachusetts Chan Medical School, Shrewsbury, MA, USA; 3 Department of Biochemistry and Molecular Biotechnology, University of Massachusetts Chan Medical School, Worcester, MA, USA; 4 Institute for Memory Impairments and Neurological Disorders, University of California, Irvine, CA, USA; 5 Department of Pharmacology, Physiology & Biophysics, Boston University Chobanian & Avedisian School of Medicine, Boston, MA, USA; 6 Bioinformatics Program, Boston University Faculty of Computing & Data Sciences, Boston, MA, USA; 7 Department of Genetics and Genomic Sciences, Icahn School of Medicine at Mount Sinai, New York, NY, USA; 8 Hope Center for Neurological Disorders, Knight Alzheimer’s Disease Research Center, Washington University School of Medicine, St. Louis, MO, USA

## Abstract

The apolipoprotein E ε4 (*APOE4*) allele is the strongest genetic risk factor for late-onset Alzheimer’s disease (AD), yet the mechanistic underpinnings by which it alters human microglial function remain poorly understood. Here, we utilize CRISPR/Cas9-engineered isogenic human induced pluripotent stem cell-derived microglia-like cells (iMGs) expressing *APOE3* or *APOE4* to systematically interrogate genotype-dependent effects on microglial proteomes and their secreted extracellular vesicles (EVs) under basal and fibrillar amyloid-β (fAβ)-challenged conditions. Quantitative proteomics revealed that *APOE4* iMGs exhibit downregulated RNA processing, impaired phagocytosis, and exacerbated pro-inflammatory signaling, particularly upon fAβ exposure. Functionally, *APOE4* iMGs demonstrated increased NF-κB activation, enhanced neuronal apoptosis and tau pathology in 2D and 3D co-culture systems. Interestingly, *APOE4* iMGs released greater amount of EVs enriched in proteasomal components over *APOE3* iMG. Integrated proteomic analyses further highlighted *APOE4*-specific selective packaging of EV cargo linked to neuron-glia communication, inflammatory signaling, and proteostasis dysregulation, suggesting a potent, non-cell-autonomous mechanism driving neuronal damage. Consistently, these *APOE4* iMGEVs were preferentially internalized by neurons, leading to disrupted neuronal proteostasis, impaired neuronal firing, and increased tau phosphorylation, particularly under pathological stress. Notably, pharmacologic inhibition of NF-κB in *APOE4* iMGs reduced EV cargo-loading of proteasomal molecules and mitigated iMGEV-driven neuronal dysfunction and tau pathology. These findings establish a mechanistic link between *APOE4*, dysregulated microglial homeostasis, and microglial EV-mediated propagation of proteostatic dysfunction and neurodegeneration, advancing our understanding of how *APOE4* contributes to AD pathogenesis.

## Introduction

The ε4 allele of the apolipoprotein E gene (*APOE*), which translates to the APOE4 isoform, is the strongest genetic risk factor for late-onset Alzheimer disease (AD)^1-5^. Compared to common *APOE3* allele, *APOE4* confers a markedly elevated risk and drives widespread pathophysiological changes in AD, including increased amyloid deposition, amyloid plaque-associated reactivity^6^, impaired brain lipid homeostasis^7^, intensified neuroinflammation^8^ and exacerbated tau-mediated neurodegeneration^9^. APOE isoform-specific effects, especially APOE4, profoundly influence AD onset and progression, highlighting its pivotal role in diverse disease mechanisms.

Recent studies in single-cell transcriptomics have demonstrated the diverse activation states of microglia during AD progression^10-12^. Microglia, the major immune cells in the brain, exhibit heterogeneous phenotypes across disease stages, and AD risk genes such as *APOE* emerge as key modulators of disease-associated microglial (DAM) and neurodegenerative microglia (MGnD) phenotypes^13,14^. Particularly, human and mouse studies demonstrate that *APOE4* disrupts microglial compaction and clearance of Aβ plaques^15^, while increasing pro-inflammatory responses and lipid accumulation in microglia^16^. Under fibrillar Aβ challenge, *APOE4* microglia is reported to upregulate the lipid-droplet associated enzyme Acyl-CoA Synthetase Long Chain Family Member 1 (ACSL1), driving triglyceride accumulation and lipid droplet formation, and secrete neurotoxic factors that induce tau hyperphosphorylation in neurons^7^. Furthermore, a recent study suggests that *APOE4* activates a transforming growth factor-β (TGFβ)-mediated checkpoint that hinders microglia from transitioning to a fully disease-associated state^15^. This aberrant TGFβ signaling, together with other *APOE4*-linked changes, impairs microglial phagocytosis of apoptotic neurons and debris, downregulates lysosomal and complement pathways, and upregulates stress and inflammatory genes in *APOE4* microglia^15^.

Extracellular vesicles (EVs) are now emerging as critical mediators of intercellular signaling and neurodegeneration in AD^17,18^. Microglia-derived EVs can propagate pathogenic tau seeds and pro-inflammatory factors, thereby exacerbating tau pathology^19^. Indeed, pharmacological inhibition of EV release or microglial depletion halts tau spread and neurotoxicity^19^. Additionally, plaque-associated microglia exhibit increased EV secretion, further accelerating tau pathology in a humanized APP mouse model^20^. Recent findings suggest that APOE4 isoform alters EV biogenesis and composition. Specifically, human brain-derived EVs from *APOE4* carriers demonstrate reduced secretion compared to those from *APOE3* carriers^21^, with proteomic analyses revealing enrichment of proteins involved in extracellular matrix-receptor interactions, phagosome and metabolic pathways^22^. However, the specific contributions of *APOE4* versus *APOE3* in microglia-derived EVs, particularly how they influence EV content and downstream effects on AD pathogenesis, remain poorly understood.

To elucidate the mechanisms by which *APOE4* contributes to AD risk through microglial dysfunction, we employed CRISPR/Cas9-engineered isogenic human induced pluripotent stem cell (hiPSC)-derived microglia homozygous for *APOE3* or *APOE4*. We conducted global quantitative proteomic analyses of both microglia and their secreted EVs under basal conditions and following fibrillar Aβ stimulation. This comprehensive, isoform-specific interrogation revealed *APOE4*-associated impairments in RNA processing, phagocytosis, and immune activation, along with selective enrichment of proteasomal components in EVs, particularly under pathological stress. Functional validation using both 2D and 3D human iPSC-derived co-culture systems demonstrated that *APOE4* microglia induce neurotoxicity, elevated NF-κB signaling, and tau hyperphosphorylation. Moreover, *APOE4*-derived EVs impaired neuronal proteostasis and activity, further promoted tau phosphorylation, effects that were partially mitigated by pharmacologic NF-κB inhibition in *APOE4* microglia, implicating inflammatory signaling as a key driver for pathogenic EV remodeling. Together, these findings uncover key *APOE4*-related alterations in microglial homeostasis and intercellular signaling, emphasizing the importance of integrated iPSC and EV-based models to define both cell-autonomous and EV-mediated mechanisms contributing to AD risk.

## Results

### Isogenic iPSC-derived *APOE3* and *APOE4* microglia exhibit distinct proteomic signatures and *APOE4* alters microglial response to fAβ challenge

Three pairs of CRISPR-Cas9 edited isogenic human iPSC lines homozygous for *APOE3* or *APOE4* alleles (Fig. S1A-S1B; Table S1) were used and differentiated into hematopoietic stem cells (HPCs), followed by microglia-like cells (iMGs) (Fig. 1A and Fig. S1C-S1E). Immunostaining confirmed significant expression of PU.1 and TREM2 in both genotypes, indicating successful microglial differentiation (Fig. 1B). To investigate the impact of APOE isoforms on microglial proteomes under normal and disease conditions, quantitative proteomic profiling using data-independent acquisition mass spectrometry (DIA-MS) was performed on *APOE3* and *APOE4* iMGs treated with either vehicle or fibrillar β-amyloid (fAβ) (Fig. 1C).

Mass spectrometry identified between 5,000 and 7,000 proteins from iMG lysates across all conditions, with each protein detected in at least one of three technical replicates per sample (Fig. S2A; Table S2). Principal component analysis (PCA) revealed modest separation by *APOE* genotype (*APOE3* vs. *APOE4*) and clear segregation based on treatment condition (control vs. fAβ-treated) (Fig. S2B). Pearson correlation analysis further confirmed the reproducibility of the dataset, showing strong intra-group correlations among biological replicates (Fig. S2C; Table S3). Under basal conditions, *APOE4* iMGs exhibited a distinct proteomic profile compared to *APOE3* iMGs, with 17 proteins significantly upregulated and 22 downregulated based on a threshold of p < 0.05 and ∣fold change∣ > 1.2 (Table S4; Fig. 1D, top). Following Aβ exposure, direct comparison between *APOE4* and *APOE3* iMGs revealed 21 proteins significantly upregulated and 77 downregulated in *APOE4* cells (Table S4; Fig. 1D, bottom). Gene Ontology (GO) and KEGG pathway enrichment analyses showed that, at baseline, proteins upregulated in *APOE4* iMGs were predominantly involved in antigen processing, lysosomal function, and protein folding (Fig. 1E; Fig. S2D and Table S5). In contrast, under fAβ-treated conditions, proteins downregulated in *APOE4* iMGs were enriched for pathways related to RNA splicing, chromatin organization, and mitochondrial ribosome function (Fig. 1E; Fig. S2D and Table S5). Ingenuity pathway analysis (IPA) further supported activation of MHC class I-mediated antigen presentation in *APOE4* iMGs under basal conditions, while revealing suppression of RNA processing pathways and SUMOylation of RNA-binding proteins following fAβ exposure (Fig. 1F; Table S6). Together, these data suggest that *APOE4* shifts microglial homeostasis toward an antigen-presenting phenotype at rest and disrupts RNA regulatory networks in response to Aβ stimulation.

We next performed within-genotype comparisons to assess the impact of fAβ stimulation relative to baseline. *APOE4* iMGs exhibited a markedly broader proteomic response, with 152 proteins upregulated and 487 downregulated, compared to 48 upregulated and 66 downregulated in *APOE3* iMGs (Fig. 1G; Table S4). Pathway enrichment analysis indicated that fAβ-stimulated *APOE3* iMGs primarily activated pathways related to viral infection and antigen presentation (Fig. S2E; Table S7). In contrast, *APOE4* iMGs engaged distinct pathways, including lysosomal function, phagosome maturation, and spliceosome-associated processes (Fig. S2F; Fig. 1H and Table S7). Notably, suppression of RNA processing pathways was more pronounced in *APOE4* iMGs than in *APOE3* iMGs following fAβ exposure (Fig. S2E-S2F; Fig. 1H and Table S7). IPA analysis further supported these findings, revealing activation of interferon-γ signaling, Th1/Th2 pathways, and antigen presentation in *APOE3* iMGs, whereas *APOE4* iMGs showed preferential activation of cGAS-STING signaling and inhibition of RNA metabolism and splicing pathways (Fig. 1I; Table S8). These distinct molecular responses were reflected in predicted biological functions: *APOE3* iMGs were associated with immune activation, while *APOE4* iMGs were enriched for apoptosis and cell death signatures in response to Aβ stimulation (Fig. 1J; Table S8). Our results reveal an APOE isoform-specific divergence in the microglial proteome, wherein *APOE4* disrupts homeostatic functions and drives inflammatory dysregulation and RNA processing impairment in response to fAβ, potentially accelerating AD pathogenesis through both cell-autonomous and non-cell-autonomous mechanisms.

### fAβ-treated *APOE4* microglia induce neurotoxicity and tau pathology in hiPSC-derived microglia-neuron co-cultures

Our proteomic data suggested that *APOE4* may disrupt lysosomal function and phagosome maturation pathways under basal and pathological conditions (Fig. 1E and 1H). To functionally assess this, we examined the phagocytic capacity of *APOE3* and *APOE4* iMGs using automated live-cell imaging of fluorescently labeled fAβ and pre-formed tau fibrils (fTau) conjugated to the pH-sensitive dye pHrodo. This dye remains non-fluorescent in extracellular environments but fluoresces brightly in acidic compartments such as phagosomes or endosomes following internalization^23,24^. This assay provides a quantitative measure of phagocytic uptake over time rather than a qualitative assessment of representative images^25^. We observed significantly reduced internalization of pHrodo-labeled fAβ in *APOE4* iMGs compared to *APOE3* iMGs (Fig. 2A), indicating impaired phagocytic function^6,26^. To determine how combined exposure to AD-related pathological substrates influences subsequent tau phagocytosis, *APOE3* and *APOE4* iMGs were pretreated with vehicle, fAβ alone, or a combination of fAβ and fTau before challenge with pHrodo-labeled fTau. fAβ pretreatment increased subsequent fTau uptake in both genotypes, whereas combined fAβ and fTau pretreatment elicited a markedly greater phagocytic response. Nevertheless, *APOE4* iMGs consistently exhibited lower fTau uptake than *APOE3* iMGs under both priming conditions, indicating that pathological priming enhances tau phagocytosis but that this response remains attenuated in *APOE4* microglia (Fig. 2B).

To further investigate how APOE isoforms differentially influence neuronal vulnerability, we established a co-culture system pairing isogenic *APOE3* or *APOE4* iMGs with hiPSC-derived neurons (iNs) harboring the P301L Tau mutation associated with frontotemporal dementia with Parkinsonism on chromosome 17 (FTDP-17; Fig. 2C)^27^. The use of the FTDP-17 patient-derived MAPT P301L line (*APOE3/3*; Fig. S3A) is to investigate whether the *APOE* genotype of microglia modifies neuronal tau-related phenotypes in a genetically constant, tau-vulnerable recipient background. Because the MAPT P301L mutation is located in exon 10 and is therefore expressed specifically in exon-10-containing 4R tau isoforms, we examined 3R and 4R tau expression in the neuronal models used in this study. This distinction is important because conventional hiPSC-derived neurons frequently express low levels of 4R tau, whereas recent models engineered to enhance exon 10 inclusion have enabled robust seeded or cell-intrinsic 4R tauopathy phenotypes^28,29^. Immunoblotting with isoform-specific antibodies detected both 3R and 4R tau was tested in iPSC-derived neurons, including the MAPT P301L models. Quantification showed that 4R tau constituted a substantial proportion of total 3R plus 4R tau in P301L iNs (Fig. S3B). These findings demonstrate that the P301L neuronal models are not restricted to 3R tau expression and express the exon-10-containing 4R isoform required for the P301L-containing transcript. iMGs were then pre-treated with either vehicle or fibrillar Aβ, followed by a two-week co-culture with P301L Tau-expressing neurons. Immunofluorescence analysis using the microglial marker IBA1 and GFP-positive neurons, combined with 3D rendering, revealed comparable total microglial volumes across conditions. However, *APOE4* iMGs exhibited significantly reduced phagocytosis of neuronal components compared to *APOE3* iMGs, particularly in the context of fAβ exposure (Fig. 2D and 2E), confirming impaired phagocytic function in *APOE4* microglia under disease-relevant stress.

We then assessed neuronal apoptosis using activated caspase-3 as an apoptotic marker (Haney et al., 2024). Under basal conditions, no significant difference in activated caspase-3 intensity was observed between neurons co-cultured with *APOE3* and *APOE4* iMGs. However, co-culture with fAβ-treated *APOE4* iMGs markedly increased caspase-3 intensity in neurons compared to all other conditions, suggesting exacerbated neuronal apoptosis (Fig. 2F-G). In contrast, *APOE3* iMGs had only a modest, non-significant effect on caspase activation, implying a relative neuroprotective phenotype. To further assess the impact on tau pathology, we stained neurons with the AT8 monoclonal antibody to detect phosphorylated tau (pTau). No significant difference in AT8 intensity was observed between neurons co-cultured with basal *APOE3* and *APOE4* iMGs. Neurons co-cultured with fAβ-treated *APOE4* iMGs exhibited significantly higher AT8 immunoreactivity than those co-cultured with basal *APOE3* or *APOE4* iMGs. Total Tau intensity was also significantly increased in the fAβ-treated *APOE4* group compared with the corresponding untreated *APOE4* iMG co-culture group (Fig. 2H-I). These imaging findings were further evaluated by immunoblotting. AT8 levels were highest in neurons co-cultured with fAβ-treated *APOE4* iMGs and were significantly increased compared with neurons co-cultured with basal *APOE3* or *APOE4* iMGs. Total tau levels were reduced in the basal *APOE3* and *APOE4* co-culture conditions but increased following fAβ stimulation in both genotypes relative to their corresponding basal conditions (Fig. 2J). To further determine whether this *APOE4*-associated pTau response was a general consequence of microglial activation, we additionally examined LPS as a non-Aβ inflammatory stimulus. LPS-treated *APOE3* iMGs significantly increased neuronal AT8 immunoreactivity compared with untreated *APOE3* iMGs. However, LPS-treated *APOE4* iMGs did not increase AT8 relative to untreated *APOE4* iMGs and induced significantly lower AT8 immunoreactivity than LPS-treated *APOE3* iMGs (Fig. S3C). Thus, LPS did not reproduce the *APOE4*-dependent pTau response observed following fAβ stimulation. Together, these findings demonstrate that *APOE4* microglia promote neuronal apoptosis and tau pathology under fAβ challenge in a hiPSC-based co-culture system, providing functional understanding of proteomic data and highlighting a potential mechanism by which *APOE4* contributes to neurotoxicity and accelerated pTau accumulation.

### *APOE4* microglia exacerbate NF-κB activation and tau pathology in a 3D iMG-brain organoid model

To investigate how APOE isoforms modulate microglia-driven inflammatory responses in a human brain-like environment^30,31^, we established an iMG-brain organoid co-culture system. Cerebral organoids were generated from a P301L Tau iPSC line derived from a FTDP-17 patient, using a well-established differentiation protocol^32,33^. These organoids were characterized at 1, 2, 3, and 5+ months using a panel of neural and cortical markers, including NESTIN, SOX2, PAX6, TBR2, GFAP, TBR1, MAP2, SATB2, and CTIP2, confirming the regional and cellular maturation throughout the differentiation (Fig. S4A-B). As shown in Fig. 3A, isogenic *APOE3* or *APOE4* iMGs, under basal or fAβ-stimulated conditions, were co-cultured with P301L Tau mutant organoids for two weeks. CM-DiI (a lipophilic tracer) labeling and time-lapse confocal imaging demonstrated successful infiltration of iMGs throughout the organoid structure over 14 days (Fig. S4C). Immunofluorescence staining further confirmed integration of IBA1^+^ microglia not only at the organoid periphery but also within deeper interior regions (Fig. 3B).

IPA of the iMG proteomes indicated that *APOE4* iMGs, upon fAβ stimulation, exhibited activation of proteins associated with cyclic GMP-AMP synthase (cGAS), stimulator of interferon genes (STING), and nuclear factor kappa-light-chain-enhancer of activated B cells (NF-κB) signaling compared to *APOE3* iMG (Fig. 3C). Consistent with this prediction, NF-κB p65 levels were significantly increased in *APOE4* iMGs monoculture under fAβ challenge, as validated by immunoblotting across three isogenic pairs and by NF-κB p65 ELISA (Fig. 3D and E). Additional immunoblotting for phosphorylated NF-κB p65 showed the highest abundance in fAβ-treated *APOE4* iMGs with a nominally significant increase relative to basal *APOE4* iMGs (p = 0.05), whereas no corresponding change in *APOE3* iMGs was observed (Fig. S5A). In the co-culture context, immunofluorescence further revealed significantly elevated NF-κB p65 expression in *APOE4* iMGs compared to *APOE3* iMGs, particularly under fAβ-stimulated conditions (Fig. 3F). Quantification confirmed *APOE4*- and Aβ-dependent increases in both total and nuclear NF-κB p65 intensity, suggesting that *APOE4* iMGs are more susceptible to cGAS-STING-NF-κB mediated inflammatory signaling compared to *APOE3* iMGs (Fig. 3G). This microglia-specific pathway, which senses cytoplasmic DNA and drives innate immune activation, has been strongly implicated in neuroinflammation and neurodegeneration in aging and tauopathies^34,35^. Furthermore, to assess the downstream impact of elevated cGAS-STING-NF-κB signaling on neuronal tau pathology, we stained the co-cultured organoids for phosphorylated tau (pSer^202^/pThr^205^ tau; AT8) and the neuronal marker MAP2. Both fAβ-treated *APOE3* and *APOE4* iMGs significantly increased AT8 intensity in brain organoids compared to that with basal iMG groups, while MAP2 expression remained unchanged across conditions (Fig. 3H and I). Notably, *APOE4* iMGs remarkably enhanced AT8 expression in organoids compared to that without iMGs (Fig. 3H and I). In addition, we also observed that total tau intensity was modestly increased in organoids cocultured with basal *APOE3* or fAβ-treated *APOE3* iMGs relative to organoids cultured without iMGs. In contrast, no significant increase in total tau was detected in organoids co-cultured with *APOE4* iMGs under either basal or fAβ-treated conditions (Fig. S5B). These findings suggest that *APOE4*-expression microglia mount heightened NF-κB activation under Aβ stress and are associated with exacerbated tau phosphorylation in a human 3D iMG-brain organoid tauopathy model.

### *APOE4* iMG-derived EVs exhibit altered proteomic composition, particularly proteasomal components and enhanced neuronal uptake under basal and fAβ-stimulated conditions

Next, to explore the impact of *APOE* isoforms on EV biogenesis in microglia, we isolated EVs from the conditioned medium of *APOE3* and *APOE4* iMGs using sequential centrifugation followed by size-exclusion chromatography, as previously described^36^ (Fig. 4A). The size distribution of EVs derived from three pairs of isogenic iMG lines (iMGEVs) was assessed using nano-flow cytometry (NanoFCM; Fig. 4B). While the median EV diameter was comparable between *APOE3* and *APOE4* iMGEVs, *APOE4* iMGs secreted significantly more EV particles per cell than the *APOE3* iMGs (Fig. 4C).

To further characterize the molecular composition of these iMGEVs, we performed quantitative proteomic profiling under both basal and fAβ-stimulated conditions using DIA-MS, enabling a direct comparison of EV cargo between *APOE3* and *APOE4* microglia for the first time (Fig. 4D). Mass spectrometry identified between 300 and 600 proteins in iMG-derived EVs across all conditions, with each protein detected in at least one of three technical replicates per sample (Fig. S6A; Table S9). Principal component analysis (PCA) revealed distinct clustering by *APOE* genotype and treatment condition, highlighting both isoform- and stimulus-specific differences in EV proteomes (Fig. 4E). Pearson correlation analysis further confirmed the strong correlations between *APOE* genotypes (Fig. S6B; Table S10). Among the total of over 600 EV-associated proteins, we identified 61 proteins (e.g., CD9, CD63, CD81, ANXA2, and PDCD6IP) overlapping with the top 100 small EV-enriched proteins listed in the ExoCarta database^37^, and 25 proteins (ITGB2, ITGAM, LCP1 and C1QC) previously defined as iMGEV-specific in our earlier study^36^ (Fig. 4F).

We next performed differential expression analysis using volcano plots to identify significantly altered proteins in *APOE4* versus *APOE3* iMGEVs under both basal and fAβ-stimulated conditions, applying a threshold of p < 0.05 and ∣fold change∣ > 1.2. Under basal conditions, *APOE4* iMGEVs exhibited 70 significantly upregulated and 76 downregulated proteins relative to *APOE3* iMGEVs (Fig. 4G, top; Table S11). Following fAβ stimulation, 30 proteins were significantly upregulated and 19 downregulated in *APOE4* compared to *APOE3* iMGEVs (Fig. 4G, bottom; Table S11). Functional enrichment analysis revealed that proteins upregulated in *APOE4* iMGEVs under basal conditions were enriched in integrin binding and proteasome-related pathways (Fig. 4H; Table S12), while downregulated proteins were associated with intermediate filament (Fig. S6C; Table S12). Those upregulated proteins in fAβ-treated *APOE4* iMGEVs were associated with inflammatory responses, antigen presentation, and proteasome function (Fig. 4H; Table S12), while downregulated proteins were related to actin cytoskeleton (Fig. S6C; Table S12). Further pathway enrichment analysis confirmed that many of the differentially expressed proteins in *APOE4* iMGEVs, under both basal and Aβ-treated conditions, were involved in proteasomal activity and pathways implicated in neurodegenerative diseases, including amyotrophic lateral sclerosis (ALS), Parkinson’s disease (PD), and AD (Fig. 4I; Table S12). Finally, we performed IPA to predict the functional consequences of differentially expressed proteins in *APOE4* iMGEVs. The analysis revealed activation of pathways related to cellular uptake, immune response, and cell adhesion by *APOE4* iMGEVs under basal conditions, with key proteins involved in IL-4/IL-13 signaling and integrin-mediated cell surface interactions (Fig. 4J; Fig. S6D and Table S13). To assess the functional impact of these APOE isoform-specific iMGEVs on neuronal uptake, we labeled purified iMGEVs with the pH-sensitive dye pHrodo and applied an equal number of EV particles to hiPSC-derived neurons at an EV-to-cell ratio of 100:1. Particle-number-matched iMGEVs were collected from *APOE3* and *APOE4* iMGs under basal and fAβ-treated conditions. Neurons exposed to *APOE4* iMGEVs exhibited significantly greater pHrodo fluorescence than neurons exposed to the corresponding *APOE3* iMGEVs under both basal and fAβ-treated conditions (Fig. 4K-L).

We also performed within-genotype comparisons to assess the effects of Aβ stimulation on iMGEV protein profiles relative to baseline. In *APOE3* iMGEVs, fAβ treatment led to modest changes, with 9 proteins significantly upregulated and 12 downregulated (Fig. S7A; Table S11). Pathway enrichment analysis revealed that the upregulated proteins were primarily associated with the vesicle lumen, while the downregulated proteins were linked to protein folding pathways (Fig. S7B; Table S14). In contrast, fAβ stimulation had a more pronounced impact on *APOE4* iMGEVs, with 36 proteins significantly upregulated and 40 downregulated compared to untreated *APOE4* controls (Fig. S7C; Table S11). The upregulated proteins were enriched in proteasome-related functions and pathways implicated in neurodegenerative diseases, whereas the downregulated proteins were associated with complement and coagulation cascades (Fig. S7D-S7E; Table S14). A heatmap of the iMGEV proteome further illustrated distinct protein clustering across all conditions, highlighting genotype- and treatment-specific differences (Fig. S7F).

Together, these findings demonstrate that *APOE4* not only alters EV biogenesis and protein cargo but also enhances EV-neuron interactions, suggesting a potential EV-mediated pathway by which *APOE4* microglia may exacerbate neuronal dysfunction and contribute to AD pathology.

### Integrated proteomic analysis reveals both concordant and divergent regulation of cellular and EV proteins in *APOE3* and *APOE4* microglia

To investigate the relationship between microglial intracellular proteome changes and EV cargo composition, we performed integrated proteomic analysis by comparing log_2_ fold changes of proteins identified in both iMGs and their corresponding EVs. This approach enabled us to assess whether specific protein alterations were reflected similarly in EV secretion or diverged across cellular and extracellular compartments.

Under basal conditions, several proteins exhibited concordant upregulation in *APOE4* iMGs and their EVs, including the proteasomal subunit PSMA5, complement components such as C1QB and C4B, and immune regulators like ITGAM. Similarly, concordant downregulation was observed for proteins including RAB5A, VDAC1, and PRDX1 (Fig. S8A). However, certain proteins, such as PSMB2, ITGAX, APOE, SORL1 and C1QA, were selectively enriched in EVs but not in the corresponding cells, while others like DSG1 and RAB10 were exclusively elevated in iMGs. Following Aβ stimulation, proteins such as C1QB and ITGAM continued to show concordant upregulation in both *APOE4* iMGs and their EVs, whereas other proteins including APOE, PSMB2, PSMB4, and SORL1 were upregulated in EVs but not in the corresponding cells (Fig. S8B).

We also examined within-genotype changes in response to Aβ stimulation. In *APOE3* iMGs, Aβ treatment led to coordinated increases in both cellular and EV levels of proteasomal subunits, including PSMA2 and PSMA5 (Fig. S8C). In contrast, *APOE4* iMGs exhibited a greater number of discordant changes, with many proteins preferentially enriched in EVs but not in the corresponding cells. These included additional proteasomal components (e.g., PSMB1, PSMB4, PSMB6), immune-related proteins (e.g., SPP1, HLA-DRB5), and tau-modulating factors (e.g., PPARD, PLD4) (Fig. S8D). These findings support that *APOE4* promotes selective EV cargo loading under both basal and pathological conditions, particularly for proteins involved in neuronglia interactions, inflammation and proteostasis.

### *APOE4* iMG-derived EVs disrupt neuronal proteasome activity, impair neuron firing, and promote tau phosphorylation

Our proteomic analysis revealed a higher enrichment of multiple 20S proteasomal subunits, including PSMA1-6 and PSMB1-10, in *APOE4* iMGEVs compared to *APOE3* iMGEVs under basal conditions, with Aβ treatment further increasing their abundance in *APOE4* samples (Fig. 5A). We therefore selected PSMB5 and PSMA1 as representative proteasomal components for orthogonal validation at the single-EV level using super-resolution imaging (ONI Nanoimager) and nano-flow cytometry, respectively. These analyses showed a significantly higher proportion of PSMB5^+^ EVs as co-stained with EV markers (pan-tetraspanins: CD9, CD63, CD81; Fig. 5B and C) and PSMA1^+^ EVs in *APOE4* iMGEVs compared to *APOE3*, in both basal and fAβ-treated conditions (Fig. S9A and B). The proteasome is a large protein complex responsible for degrading ubiquitinated proteins and plays critical roles in neural function, brain aging, and neurodegenerative disease pathogenesis^38^.

To assess the functional consequences of altered proteasome content in iMGEVs, we treated iPSC-derived WT or P301L Tau neurons with iMGEVs collected from control or fAβ-stimulated *APOE3* and *APOE4* microglia (Fig. 5D). Proteasomal activity assays revealed that WT neurons exposed to fAβ-treated *APOE4* iMGEVs exhibited significantly reduced chymotrypsin-like activity compared to those treated with *APOE3* iMGEVs (Fig. 5E). Trypsin-like activity was also reduced in neurons treated with *APOE4* iMGEVs and with fAβ-treated EVs from either genotype (Fig. 5E), while no significant changes were observed in caspase-like activity (Fig. S9C). These data suggest that *APOE4* and Aβ exposure synergistically impair neuronal proteasome function via EV-mediated transfer of proteasome-related components.

Given that hyperphosphorylated tau contributes to neurofibrillary tangle formation and neurodegeneration in AD, and that proteasome dysfunction has been linked to tau accumulation^39^, we next evaluated whether *APOE4* iMGEVs influence tau pathology and neurotoxicity. The level of activated caspase-3 in P301L Tau iNs was evaluated after incubation with different iMGEVs. Our results revealed no difference in the activation of caspase-3 level between *APOE3* and *APOE4* iMGEV-treated groups (Fig. S9D and E). However, Tau ELISA revealed that *APOE4* iMGEVs, especially those from Aβ-treated microglia, significantly increased tau phosphorylation at the pT181 site in P301L Tau neurons (Fig. 5F), whereas total tau and pS396 tau levels remained unchanged (Fig. S9F). In contrast, *APOE3* iMGEVs showed a mild, non-significant reduction in total tau and pS396 tau levels compared to the vehicle-treated group (Fig. S9F). These results indicate that *APOE4* microglial EVs may exacerbate tau pathology by promoting hyperphosphorylation of tau, with a preferential increase in pT181 tau among the tau measures examined.

We next assessed the effect of iMGEVs on neuronal network activity by using calcium imaging and high-density multi-electrode array (HD-MEA) recordings. WT neurons infected with lentivirus expressing the GCaMP6-mRuby calcium indicator were treated with iMGEVs, and calcium activity was monitored longitudinally using an IncuCyte S3 live-cell imaging system (Fig. 5G). Quantitative analysis over five days revealed that fAβ-treated *APOE4* iMGEVs significantly disrupted neuronal activity, as evidenced by reduced burst duration and burst strength, and increased burst rate (Fig. 5H). Moreover, neurons treated with *APOE3* iMGEVs showed enhanced synchronization by day 5, as indicated by increased network mean correlation (Fig. 5H), suggesting a potential beneficial role for *APOE3* iMGEVs in promoting neuronal network. Complementary HD-MEA analysis further confirmed that exposure to fAβ-treated *APOE4* iMGEVs reduced electrophysiological output, including decreases in mean firing rate and spike amplitude over sequential recording days (Fig. 5I-J). Interestingly, although exposure to fAβ-conditioned *APOE3* iMGEVs also reduced spike amplitude during the post-treatment period, it transiently increased mean firing rate relative to the pretreatment baseline (Fig. S10A). This response contrasted with the reduction in mean firing rate observed following exposure to fAβ-conditioned *APOE4* iMGEVs, indicating divergent early effects of *APOE3* and *APOE4* iMGEVs on neuronal firing. Together, these findings support a mechanism by which *APOE4*-expressing microglia, particularly under fAβ-stimulated conditions, contribute to neuronal dysfunction and tau pathology through EV-mediated transfer of proteostasis-related cargo.

### NF-κB inhibition in *APOE4* microglia attenuates their proteasome-enriched EV release and mitigates microglial EV-driven neuronal dysfunction and tau pathology

Microglial NF-κB signaling has been implicated as a key driver of tau pathology propagation and neurotoxicity in tauopathy models, where NF-κB inhibition reduces the release of phosphorylated tau and attenuates tau spreading^40^. Due to the observation of heightened NF-κB activation in fAβ-stimulated *APOE4* iMGs, we next tested whether pharmacologic NF-κB inhibition could mitigate *APOE4*-dependent EV remodeling and downstream neuronal dysfunction. *APOE4* iMGs were treated with vehicle (DMSO) or the NF-κB inhibitor TPCA1 during basal or fAβ-stimulated conditions, followed by EV collection and functional examinations in recipient neurons (Fig. 6A). Single-EV imaging confirmed that fAβ increased the proportion of PSMB5^+^ EVs from *APOE4* iMGs, whereas TPCA1 treatment markedly reduced the PSMB5^+^ EV population and blunted the fAβ-associated increase (Fig. 6B-C), indicating that inflammatory signaling contributes to proteasome-associated EV cargo loading and may regulate EV subtype release.

Moreover, our results showed that TPCA1 pretreatment of *APOE4* iMGs partially restored neuronal proteasomal activity. iPSC-neurons treated with EVs from DMSO-conditioned *APOE4* iMGs exhibited significantly reduced chymotrypsin-like and trypsin-like proteasomal activities relative to vehicle-treated neurons. In contrast, proteasomal activities in neurons treated with EVs from TPCA1-conditioned *APOE4* iMGs were not significantly different from vehicle and showed a numerical shift toward vehicle levels. However, direct comparisons between the corresponding DMSO- and TPCA1-conditioned EV groups did not reach statistical significance, indicating a partial attenuation of the EV-associated proteasomal deficit (Fig. 6D). In parallel, TPCA1-conditioned *APOE4* iMGEVs reduced tau phosphorylation burden in recipient P301L tau neurons, as revealed by decreasing the elevation of pT181 tau/total tau induced by *APOE4* EV exposure, with minimal effects on total tau levels (Fig. 6E). Finally, longitudinal calcium imaging demonstrated that TPCA1 also mitigated *APOE4* EV-driven neuronal activity defects, mainly improving burst duration and strength compared with neurons treated with EVs from DMSO-conditioned *APOE4* iMGs under basal and fAβ-stimulated conditions (Fig. 6F-G). In the *APOE3* background, DMSO-conditioned EVs produced only modest effects; however, fAβ stimulation reduced burst duration, an effect attenuated by TPCA1, which also increased burst strength (Fig. S10B). Our findings indicate that NF-κB signaling in donor microglia modulates EV effects on neuronal network activity across *APOE* genotypes, with outcomes dependent on both genotype and activation state.

Collectively, these data support a mechanistic link between *APOE4*-associated inflammatory activation, particularly NF-κB signaling, and the generation of proteasome-enriched, neurotoxic microglial EVs, and further indicate that dampening NF-κB activity in *APOE4* microglia can partially uncouple EV cargo dysregulation from downstream neuronal proteostasis impairment and tau pathology (Fig. 6H).

## Discussion

Increasing evidence underscores the complexity of microglial responses in AD, with substantial modulation driven by *APOE* genotype^11,16,41^. In this study, we employed the iPSC-derived microglia model to comprehensively investigate the proteomic landscapes of isogenic *APOE3* and *APOE4* microglia under both basal conditions and following fAβ stimulation, as well as the proteomes of their corresponding EVs for the first time. Through quantitative proteomic analyses, we identified extensive *APOE* isoform-dependent alterations in both cellular and EV-associated protein signatures. Importantly, we uncovered a previously unrecognized mechanism by which *APOE4* microglia may promote tau pathology and disease propagation via EV-mediated delivery of proteasomal components that may impair neuronal proteostasis and exacerbate tau pathology.

Under basal conditions, *APOE4* iMGs exhibited subtle proteomic changes compared to their *APOE3* counterparts, with a limited subset of differentially expressed proteins enriched in pro-inflammatory and antigen-presentation pathways, including upregulation of MHC class I molecules (e.g., TAP1 and TAP2). This shift from resting microglia into immune-active by *APOE4* genotype is consistent with previous studies^6,13^. However, upon exposure to fAβ, the divergence between *APOE3* and *APOE4* microglia became more pronounced. While fAβ-treated *APOE3* iMGs mount a robust immune response characterized by activation of inflammatory signaling and enhanced antigen presentation, *APOE4* iMGs exhibit significant downregulation of proteins involved in RNA processing and splicing, including reduced SUMOylation of RNA-binding proteins as showed by IPA. These findings align with recent single-nucleus RNA sequencing studies that reported reduced ribosomal and antigen-presenting response (HLA) clusters in *APOE4* microglia in response to Aβ pathology^41,42^. SUMOylation, a post-translational modification that modulates the activity and localization of RBPs, plays a crucial role in RNA metabolism and stress responses^43^. Disruptions in this pathway have been implicated in neurodegenerative diseases and may compromise key regulatory mechanisms that influence microglial function and neuroinflammation^43-45^. We speculate that the loss of RNA homeostasis, potentially mediated by impaired SUMOylation, could also contribute to the broader dysfunction of *APOE4* microglia.

Future studies are warranted to elucidate the mechanistic links between SUMOylation impairment and RNA dysregulation in *APOE4* genotype, which may reveal novel therapeutic targets to restore microglial function in AD.

Previous studies have also linked the APOE4 isoform to impaired phagocytic capacity in microglia^6,10,42,46^. Consistent with these findings, we functionally validated that *APOE4* iMGs exhibited significantly reduced uptake of both fibrillar Aβ and tau aggregates. Notably, we demonstrated for the first time that fAβ stimulation enhances fTau uptake in both *APOE3* and *APOE4* microglia *in vitro*, suggesting that fibrillar Aβ may serve as a priming signal to increased microglial clearance of tau under disease-relevant conditions. However, this response was markedly attenuated in *APOE4* iMGs, which displayed significantly lower fTau internalization following Aβ exposure. These results indicate that Aβ-induced activation is compromised in *APOE4* microglia, potentially leading to impaired tau clearance and contributing to Aβ-induced tau pathology^9,47^.

These phenotypes were further recapitulated in our human iPSC-based neuron-microglia and microglia-organoid co-culture models. In 2D co-cultures, fAβ-challenged *APOE4* iMGs exhibited reduced phagocytosis of neuronal compartments, increased caspase-3 activation and elevated AT8-positive tau pathology. In our 3D organoid co-culture system, we observed significant activation of the NF-κB signaling pathway and elevated tau phosphorylation specifically in *APOE4* microglia following Aβ exposure. Recent studies have demonstrated that cGAS-STING signaling in microglia, triggered by cytosolic DNA or damaged organelles, drives age-related inflammation and neurodegeneration^34,35,48^. Consistently, IPA of our iMG proteomic data indicated stronger activation of apoptosis- and organismal death-associated pathways in fAβ-treated *APOE4* iMGs than in the corresponding *APOE3* condition. Notably, the combination of APOE4 and TREM2 R47H has been shown to amplify tauopathy-induced microglial cGAS-STING signaling and downstream interferon response^48^. In our study, proteomic pathway analysis implicated cGAS-STING-associated inflammatory signaling in *APOE4* iMGs, whereas complementary biochemical and imaging analyses provided more direct evidence of enhanced NF-κB signaling. Specifically, *APOE4* iMGs exhibited increased total and nuclear NF-κB p65 in the FTDP-17 patient-derived organoid co-culture model, and phosphorylated NF-κB p65 was highest in fAβ-treated *APOE4* iMG monocultures, with a nominally significant increase relative to basal *APOE4* iMGs. These findings are consistent with heightened NF-κB-associated inflammatory responsiveness in *APOE4* microglia under disease-relevant stress and suggest a potential link to the accompanying increase in neuronal tau pathology. However, because direct activation of upstream cGAS-STING components was not established biochemically, the involvement of this pathway remains suggestive and will require activation-specific assays and targeted perturbation in future studies. Additionally, our results align with previous studies demonstrating that *APOE4* microglia exaggerated neurotoxicity^7^. Although our proteomic analysis did not reveal clear evidence of lipid-associated dysfunction, such as the formation of lipid-droplet-accumulating microglia previously described^7,49,50^, it remains possible that underlying lipid metabolic abnormalities may contribute to the observed neurotoxicity and tau pathology.

One of the new aspects of our study is the comprehensive proteomic profiling of EVs secreted by isogenic *APOE3* and *APOE4* iMGs, performed under both basal and Aβ-stimulated conditions using DIA-MS. While prior studies have suggested that the *APOE4* genotype alters EV biogenesis and cargo composition^21,22^, our dataset represents the first in-depth quantitative analysis of microglia-derived EVs from a human isogenic model, providing unprecedented insights into genotype- and pathology-specific changes. We found that *APOE4* microglia secreted significantly more EVs than *APOE3* microglia, with notable enrichment in integrin-binding proteins and proteasomal components. The increased neuronal uptake of *APOE4* iMGEVs may be partially attributed to the enhanced presence of surface integrins, which are known to facilitate EV internalization by recipient cells^51^. Moreover, disease-associated microglial proteins, including APOE and ITGAX^14^, were highly enriched in *APOE4* iMGEVs, suggesting a potential role for microglial EVs in regulating disease-associated microglial phenotypes in *APOE4* background. Notably, APOE has also been shown to facilitate β-amyloid fibrillization through interactions within EVs, as demonstrated by van Niel et al.^52^. These results are particularly relevant given growing recognition of EVs as key mediators of microglia-neuron communication, trans-cellular tau propagation, and neuroimmune signaling in AD^19,20,53^.

Another particularly intriguing finding is the selective enrichment of proteasomal subunits in *APOE4* iMGEVs, particularly following Aβ exposure. Although the upstream mechanisms governing proteasome cargo enrichment are not fully defined, our results now provide functional evidence implicating NF-κB activity as a potential regulator of this axis. Pharmacologic inhibition of NF-κB signaling in *APOE4* microglia reduced the abundance of proteasome-associated EV signatures (e.g., PSMB5^+^ EV population) and partially ameliorated neuronal dysfunctions upon *APOE4* EV exposure in basal and fAβ-treated conditions, including proteasome activity deficits, tau phosphorylation and impaired neuronal activity. Elevated inflammatory signaling, such as NF-κB activation, is known to impair proteostasis by disrupting proteasome activity^54-56^. Under these conditions, EVs may serve as a cellular mechanism to selectively package and export damaged or surplus proteasomal proteins, reflecting a maladaptive attempt to mitigate intracellular proteotoxic stress. The ubiquitin-proteasome system (UPS) is a vital regulator of neuronal proteostasis, responsible for degrading misfolded and aggregated proteins including hyperphosphorylated tau and Aβ peptides^57^. Dysfunction of the UPS is a well-established contributor to neurodegenerative diseases, with evidence showing that proteasome impairment leads to tau stabilization and aggregation into neurofibrillary tangles^39,58^. While microglia-derived EVs have been previously implicated in the intercellular transmission of pathological tau^19^, our data suggest a more direct mechanism by which *APOE4* iMGEVs actively disrupt neuronal proteostasis. EVs enriched in proteasomal subunits may interfere with the endogenous UPS machinery in recipient neurons, impairing the clearance of pathological proteins and thereby accelerating tau pathology ^39^. Indeed, we observed that *APOE4* iMGEVs, especially those derived from fAβ-challenged microglia, significantly impaired neuronal proteasomal activity, elevated phosphorylated tau (pThr^181^) levels and reduced neuron firing in recipient neurons. Recent human iPSC-based evidence revealed that microglia-derived conditioned media are sufficient to reshape neuronal network dynamics, and that inflammatory *APOE4* microglia drive stronger changes in neuronal circuit activity than *APOE3*^49,59^. Our results further identify a novel *APOE4*-dependent, EV-mediated mechanism of neuronal dysfunction, wherein microglial EVs act as active messenger for propagating proteotoxic stress and tau pathology. Notably, the proteomic divergence between *APOE4* iMGs and their corresponding EVs, particularly the selective enrichment of proteasome-related proteins in EVs but not in parental cells, suggests that cargo sorting into EVs is not passive but regulated in an *APOE* genotype-dependent manner. This active, isoform-specific EV packaging may represent a previously unrecognized mechanism of non-cell-autonomous neurodegeneration in AD. Future studies combining targeted and combinatorial manipulation of EV cargo components will be needed to define their individual and cooperative contributions to neuronal proteostasis and tau pathology. More broadly, elucidating how inflammatory signaling intersects with proteasomal regulation and EV biogenesis in *APOE4* microglia may reveal cell-type-specific mechanisms that drive the propagation of neurodegenerative pathology.

Despite these advances, our study has several limitations. First, while the use of an isogenic system offers a strong advantage in controlling for inter-individual genetic variability, the proteomic analyses were conducted using three pairs of isogenic *APOE3* and *APOE4* iPSC-derived microglial lines, which limits statistical power for protein-level differential-abundance testing after correction for multiple comparisons. We therefore report nominal values and interpret nominally associated proteins as hypothesis-generating candidates. The principal biological conclusions are based on coordinated protein and pathway patterns together with orthogonal single-EV, biochemical, and functional validation. Nevertheless, larger independent hiPSC cohorts will be required to confirm individual protein-level associations and assess their generalizability. Additionally, although the isogenic design mitigates donor variability, it does not capture individual-specific variation or the full spectrum of *APOE4* haplotypes as one of the study lines is derived from *APOE3* haplotype^10^. Future studies incorporating a broader panel of donor-derived hiPSC lines stratified by *APOE* genotype, haplotype, and clinical background will improve generalizability and allow for a more comprehensive investigation of gene-environment interactions. Finally, while our *in vitro* systems capture key aspects of microglia-neuron and EV-neuron interactions, they cannot fully recapitulate the complexity of the *in vivo* brain microenvironment, including vasculature, peripheral immune inputs, and chronic pathology. *In vivo* validation of our findings, particularly of the NF-κB-EV-proteostasis axis in *APOE4* background and its contribution to tau pathology will be important to establish translational relevance.

In summary, our study provides integrated and new insights into *APOE* isoform-specific regulation of microglial function and EV-mediated intercellular communication. These findings emphasize microglia as well as microglial EVs as key mediators of *APOE4*-associated neurodegeneration and targeting inflammatory signaling pathways that govern EV cargo remodeling may be a tractable strategy for therapeutic intervention in neurodegenerative diseases.

## Materials and Methods

### Human induced pluripotent stem cell (hiPSC) cultures

Three pairs of isogenic hiPSC lines carrying either the *APOE3/3* or *APOE4/4* genotype were used in this study. Among them, one pair of isogenic line was obtained from ALSTEM (Catalog# iPS26 and iPS16), and the other two pairs were provided by the University of California, Irvine Alzheimer’s Disease Research Center (UCI ADRC; Table S1) ^10^. The *APOE* genotype was confirmed by Sanger sequencing with the forward primer 5’-GGCACGGCTGTCCAAGGA-3’ and reverse primer 5’-GCCCCGGCCTGGTACAC-3’. The G-banding karyotyping of all hiPSC lines was performed by WiCell Cytogenetics (Madison, WI) to validate the normality of chromosomes. Pluripotency of all lines was confirmed using the Human Pluripotent Stem Cell Marker Antibody Panel (R&D Systems, Cat# SC008), including antibodies against OCT3/4, NANOG, alkaline phosphatase, and SSEA-4, following the manufacturer’s protocol. Cells were cultured under feeder-free conditions on Matrigel-coated plates (Corning, Cat# 354277) in mTeSR Plus medium (StemCell Technologies, Cat# 100-0276) and maintained in a humidified incubator at 37°C with 5% CO_2_. Media was changed every other day, and cells were passaged every five days. All lines were authenticated and confirmed to be mycoplasma-free.

### Generation of isogenic *APOE* hiPSC lines

Two *APOE* isogenic pairs (RC1C/CD1F and MC2E/RC2I; Tabe S1) were generated via nucleofection of Cas9-sgRNA RNP complexes as previously described^10^. Briefly, the sgRNA and ssODN repair template were designed using an online guide-design tool (https://www.deskgen.com/guidebook/). The ssODN contained the nucleotide changes required to convert the *APOE* genotype, together with silent mutations that disrupted the PAM sequence and removed a NotI restriction site to facilitate prevention of repeated Cas9 cleavage and clone screening. sgRNA activity was first validated after nucleofection of hiPSCs with Cas9-sgRNA ribonucleoprotein complexes. For isogenic line generation, hiPSCs were nucleofected with preassembled Cas9-sgRNA complexes and ssODN repair template. After 48 hours, single SSEA4-positive cells were isolated by FACS into Matrigel-coated 96-well plates and clonally expanded. Candidate clones were screened by PCR and loss of the NotI restriction site, followed by TaqMan SNP genotyping and Sanger sequencing to confirm homozygous *APOE* editing.

### Generation of hiPSC-microglia-like cells (iMGs)

hiPSCs were differentiated to hematopoietic progenitor cells (HPCs) and subsequently to microglia as previously described with minor modifications^36,60^. Briefly, hiPSCs colonies were plated at low density and differentiated into hematopoietic progenitor cells (HPCs) using the STEMdiff Hematopoietic Kit (StemCell Technologies, Cat# 05310). After 10-12 days, non-adherent HPCs emerged in the culture medium, were gently collected, and replated onto Matrigel-coated 6-well plates at a density of 200,000 cells per well for microglial differentiation. To generate iMGs, HPCs were cultured in microglia basal medium composed of DMEM/F12 (Cat# 11330032), 2× insulin-transferrin-selenite (Cat# 41400045), 1× Glutamax, 1× non-essential amino acids (NEAA), 2% B27, 0.5% N2 (Cat# 17502048, all from Invitrogen), 400 μM monothioglycerol (Sigma-Aldrich, Cat# M1753), and 5 μg/mL additional insulin (Sigma-Aldrich, Cat# I2643). This medium was supplemented with 50 ng/mL TGF-β1 (Peprotech, Cat# 100-21), 25 ng/mL M-CSF (Cat# 300-25), and 100 ng/mL IL-34 (Cat# 200-34, Peprotech). After 25 days of differentiation, iMGs were further matured for 3 days by additional supplement with 100 ng/mL CD200 (R&D Systems, Cat# 2724-CD-050) and 100 ng/mL CX3CL1 (Peprotech, Cat# 300-31). iMG identity was confirmed by immunocytochemistry for microglia-specific markers.

### Generation of induced hiPSC-neurons (iNs) by *NGN2* overexpression

Rapid induction of hiPSC into human excitatory neurons was performed via a doxycycline-inducible neurogenin-2 (*NGN2*) system, as previously described^61,62^. The lentiviral construct pLV-TetO-hNGN2-eGFP-Puro (Addgene plasmid #79823, gift from Kristen Brennand) and the reverse tetracycline transactivator vector FUdeltaGW-rtTA (Addgene plasmid #19780, from Konrad Hochedlinger) were used to deliver the NGN2 system. Lentiviruses were produced in HEK293T cells (ATCC, Cat# CRL-3216) following established protocols^63^. On day −1, iPSCs were dissociated into single cells using Gentle Cell Dissociation Reagent (STEMCELL Technologies, Cat# 100-0485) and plated at a density of 50,000 cells/cm^2^. Lentiviral transduction was performed on day 0 in fresh mTeSR plus medium. *NGN2* expression was induced on day 1 (D1) by adding doxycycline (2 μg/mL) in differentiation medium composed of KnockOut DMEM (Cat# 10829018), KnockOut Serum Replacement (Cat# 10828010), MEM-NEAA (Cat# 11140050), Glutamax (Cat# 35050061), and β-mercaptoethanol (Cat# 21985023; all from Invitrogen). Puromycin (5 μg/mL) was added on day 2 (D2) for selection. On day 4 (D4), cells were replated into 96-well plates (20K cells/well) or 6-well plates (1 million cells/well) pre-coated with poly-L-ornithine (10 μg/mL; Sigma-Aldrich, Cat# P4957) and mouse laminin (5 μg/mL; Invitrogen, Cat# 23017015). Cells were maintained in neuronal maturation medium consisting of Neurobasal (Gibco, Cat# 21103049), B27 (Gibco, Cat# 17504044), Glutamax, 20% dextrose (Sigma-Aldrich, Cat# G8769), MEM-NEAA, and supplemented with 10 ng/mL each of BDNF (Cat# 450-02), GDNF (Cat# 450-10), and CNTF (Cat# 450-13; all from PeproTech). Half of the culture medium was replaced every three days. On days 14 and 21, neuronal identity was confirmed by immunocytochemistry for excitatory neuron-specific markers. In this study, we used two types of iPSC-derived neurons bearing *APOE3/3* genotype: iP26 is derived from a healthy individual (WT iNs) and the other one (MC0070) is derived from a frontotemporal dementia patient carrying the MAPT P301L mutation (P301L Tau iNs) collected by Mayo iPSC core facility. For organoids, P301L Tau mutant line was used.

### Generation of human brain organoids

3D neural spheroids were generated from hiPSCs following a previously described protocol with minor modifications^32^. Briefly, hiPSCs were dissociated into single cells using Gentle Cell Dissociation Reagent (STEMCELL Technologies, Cat# 100-0485) at 37°C for 6 min. To generate uniformly sized spheroids, 3 × 10^6^ single cells were seeded into AggreWell 800 plates (STEMCELL Technologies, Cat# 34815), which contain 300 microwells per well, in mTeSR Plus medium supplemented with 10 μM ROCK inhibitor Y-27632 (Selleckchem, Cat# S1049). Plates were centrifuged at 100 × g for 3 min to facilitate cell capture in microwells, followed by incubation at 37°C with 5% CO_2_. After 24 h, spheroids were collected by pipetting and transferred into ultra-low attachment 10-cm dishes (Corning, Cat# 3262) containing hiPSC KSR medium (comprising KnockOut DMEM, KnockOut Serum Replacement, MEM-NEAA, and β-mercaptoethanol) supplemented with two SMAD pathway inhibitors: dorsomorphin (2.5 μM, Sigma, Cat# P5499) and SB-431542 (10 μM, Tocris, Cat# 1614). From day 2 to day 6, medium was changed daily with continued dorsomorphin and SB-431542 supplementation. On day 6, spheroids were transitioned to neural induction medium containing Neurobasal-A (Thermo Fisher Scientific, Cat# 10888-022), B-27 supplement minus vitamin A (Cat# 12587010), and GlutaMAX (1:100; Cat# 35050-061). From day 6 to day 24, the medium was supplemented with 20 ng/mL epidermal growth factor (EGF; Peprotech, Cat# AF-100-15) and 20 ng/mL basic fibroblast growth factor (bFGF; Peprotech, Cat# 100-18B). Medium was changed daily for the first 10 days, then every other day for the next 9 days. To promote neuronal differentiation, from day 24 onward, the medium was supplemented with 20 ng/mL brain-derived neurotrophic factor (BDNF; Peprotech, Cat# 450-02) and 20 ng/mL neurotrophin-3 (NT-3; Peprotech, Cat# 450-03), with medium changes every other day. From day 43, spheroids were maintained in neural medium without added growth factors, with medium changes every four days.

### Cryopreservation

Spheroids were harvested and fixed in 4% paraformaldehyde (PFA) at 4°C overnight. Following fixation, samples were washed in PBS and cryoprotected in 30% sucrose solution for 48 h at 4°C. The spheroids were then embedded in Tissue-Tek optimum cutting temperature (OCT) compound (Sakura Finetek, Cat# 4583), snap-frozen on dry ice, and stored at −80°C until further processing. For immunohistochemical analysis, 12-μm-thick cryosections were prepared using a cryostat (Leica).

### Fibrillar Aβ (fAβ) and LPS treatment on iMG

On day 28 of differentiation, mature iMGs were treated with either vehicle (PBS) or human synthetic amyloid beta 1-42 pre-formed fibrils (fAβ; Cat# AF-1002-2, rPeptide or Cat# SPR-487B, StressMarq Biosciences) at a final concentration of 1 μg/mL for three days to induce a disease-associated microglial response^64^. Prior to treatment, fAβ was sonicated using an ultrasonic sonicator (Branson CPX2800H) at highest level for 10 min to fragment the fibrils. During the treatment period, cultures were supplemented with 100 ng/mL IL-34 and 10 ng/mL GM-CSF (R&D Systems, Cat# 215-GM-010/CF) to further enhance microglial activation in vitro, as previously described ^65^. Following treatment, conditioned media were collected for EV isolation, and cells were either lysed or fixed for downstream analyses.

For LPS stimulation, mature iMGs were treated with LPS at a final concentration of 1 ng/mL for 6 h. Following LPS treatment, *APOE3* and *APOE4* iMGs were co-cultured with iNs for 14 days to evaluate the effects of a non-Aβ inflammatory stimulus on neuronal tau phosphorylation. At the end of the co-culture period, cells were fixed and processed for immunofluorescence staining and AT8 quantification.

### TPCA1 treatment on iMG

IκB kinase inhibitor TPCA-1 (Cat# 2559, Tocris) was diluted in DMSO with stock concentration 1mM. Mature *APOE3* or *APOE4* iMGs were pretreated with vehicle (DMSO) or the NF-κB inhibitor TPCA1 (1:10000; final concentration 0.1μM) for 2 h, followed by exposure to control or fAβ conditions as mentioned above. Microglial EVs were then isolated and applied to WT or P301L Tau iNs for downstream analyses.

### Establishment of iMG/induced neurons and iMG/organoid co-culture systems

For the iMG-iN co-culture, hiPSC-derived neurons were seeded into glass-bottom 96-well plates (Cellvis, Cat# P96-1.5H-N) at a density of 20,000 cells per well. On day 9 of neuronal differentiation (D9), 10,000 isogenic iMGs from either under basal conditions or pre-stimulated with fAβ were added per well. The co-cultures were maintained for two weeks, after which cells were either fixed or harvested for downstream analyses.

For the iMG-organoid co-culture, 100,000 isogenic iMGs (basal or fAβ-stimulated) were added to each mature brain organoid (~6 months old). Following a two-week incubation, co-cultured organoids were either fixed or harvested for further analysis.

### Immunofluorescent (IF) staining

For the cultured cells, samples were washed three times with 1 × Dulbecco’s phosphate-buffered saline (DPBS; Thermo Fisher Scientific, Cat# 14190144) and fixed in 4% PFA at 37°C for 15 min. For human organoid sections, antigen retrieval was performed in sodium citrate for 20 mins at 95°C and rinsed 2 times with PBS. The samples were permeabilized and blocked in a blocking solution [1 × PBS, 5% bovine serum albumin (BSA, Sigma-Aldrich, Cat# A7906), 5% goat (Thermo Fisher Scientific, Cat# 16210-064) or donkey serum (Sigma-Aldrich, Cat# D9663), and 1% Triton X-100 (Sigma-Aldrich, Cat# T9284)] at room temperature (25°C) for 1 h. They were then labelled overnight at 4°C and the primary antibodies used for hiPSCs were anti-OCT3/4, anti-NANOG, anti-alkaline phosphatase, and anti-SSEA-4 (all 1:100); the hiPSC-microglia-like cells were anti-Triggering Receptor Expressed On Myeloid Cells 2 (TREM2, 1:1000, R&D, Cat# AF1828), anti-PU.1 (1:1000, Cell Signaling Technology, Cat# 2258S), and anti-IBA1 (1:500, Cat# 019-19741, Wako Chemicals); the hiPSC-neurons were anti-MAP2 (1: 200, Cat# 13-1500, Thermo Fisher Scientific), anti-Ac-Caspase 3 (1:1000, Cell Signaling Technology, Cat# 9661S), anti-pTau (AT8, Cat# MN1020, Thermo Fisher Scientific); the organoids were anti-paired Box 6 (PAX6, 1:1000, R&D, Cat# AF8150), anti-SRY-Box transcription factor 2 (SOX2, 1:1000, R&D, Cat# AF1828), and anti-NESTIN (1:1000, R&D, Cat# MAB1259), anti-TBR2 (1:200, Abcam, Cat# ab23345), anti-TBR1 (1:200, Abcam, Cat# ab31940), anti-SATB2 (1:200, Abcam, Cat# ab92446), anti-CTIP2 (1:200, Abcam, Cat# ab18465), anti-glial fibrillar acidic protein (GFAP, 1:700, Agilent, Cat# Z033429), anti-RNA binding Fox-1 homolog 3 (NeuN, 1:50, Chemicon, Cat# MAB377B), anti-MAP2 (1: 200, Cat# 13-1500, Thermo Fisher Scientific), anti-NF-kB-p65 (1: 20, Cat# sc8008, Santa Cruz Biotechnology). Alexa Fluor secondary antibodies (Life Technologies) were conjugated to the target species at room temperature (25 °C) for 1 h in blocking buffer. DAPI (4’,6-diamidino-2-phenylindole, 1:2500, Thermo Fisher Scientific, D1306) was used to label the nuclei. Images were acquired using a Leica SP8 confocal microscope (Leica).

### Isolation of iMG-derived extracellular vesicles (iMGEVs)

iMGs were plated in a 6-well plate at a density of 1 × 10^6^ cells/well, and media from D31-38 under basal or fAβ-treated conditions were collected for iMG-EV isolation. All fresh media used were ultra-centrifuged at 110,000 × g for 18 h at 4°C (Beckman Coulter, Brea, CA) to remove exogenous exosomes and other extracellular vesicles. iMGEVs were isolated by differential centrifugation, as previously described with minor modifications ^36^. First, the medium was centrifuged at 300 × g for 10 min at room temperature (25 °C) to remove floating cells. The remaining supernatant was centrifuged at 2,000 × g for 10 min at 4°C to remove cell debris, sequentially 5,000 × g for 30 min at 4°C to remove large microvesicles and apoptotic bodies, and then ultra-centrifuged at 110,000 × g overnight at 4°C in a 41Ti rotor (Optima-XE SW41, Beckman Coulter) to obtain small EV pellets. The final pellets were resuspended in 1 mL cold PBS and loaded on qEVoriginal 70nm size exclusion chromatography columns (IZON Science) to harvest fractions (500 μL each) according to the manufacturer’s instructions. Each fraction was analyzed using nano-flow cytometry analysis to determine the pooled EV-enriched fractions (f7-11). The pooled fractions were concentrated with Amicon Ultra centrifugal 100 kDa filters (Millipore, Cat# UFC510008) to a final volume of 250 μL.

For downstream EV treatment analyses, EVs were pooled from three isogenic iMGs of the same *APOE* genotype (*APOE3* or *APOE4*) and treatment condition (basal or fAβ-treated, DMSO or TPCA-1 treated) to generate representative samples for each group.

### Super-resolution imaging analysis (ONI Nanoimager)

A temperature-controlled Nanoimager S Mark II super-resolution microscope equipped with a 100×, 1.4NA oil immersion objective from ONI (Oxford Nanoimaging, Oxford, UK) was used to capture single molecules in EVs. We followed an established protocol^66,67^ with minor modifications. Briefly, the cover glasses were coated with 8-methoxypyrene-1,3,6-trisulfonic acid, trisodium salt (MPTS, Cat# M395, Fisher Scientific), dissolved at 6% in ethanol, for 30 minutes. The glasses were then incubated with Sulfo-GMBS (10 μM in PBS, Cat# PI22324, Fisher Scientific) for another 30 min. After treatment, the glasses were incubated with human TIM4/Human Fc Chimera recombinant protein (1 mg/mL, Cat# 081-10261, Fujifilm) overnight at 4°C. A 3 μL volume of the iMGEV sample (5 × 10^9^ particles/mL) was mixed with 0.3 μL 0.1% triton and incubated with primary antibodies diluted in 1.7 μL blocking buffer overnight at 4°C. The fluorescent antibodies included CL488 conjugated anti-PSMB5 (1:1000, Cat# CL488-67959, Proteintech), a pan-tetraspanin antibody cocktail consisting of anti-human CD9-APC (1:100, Cat# 341648, BD biosciences), anti-human CD81-APC (1:100, Cat# 349509, BioLegend), and anti-human CD63-APC (1:100, Cat# A15712, Thermo Fisher Scientific). Only the dye without the EV sample was used to test quality and dilution for the fluorescence antibody. The stained EVs were then treated with 1 μL of CaCl2 (1 μM) and pipetted onto the pre-coated cover glasses for attachment over 1 hour. Following PBS washes, the fixation buffer (4% PFA + 4% sucrose) was applied, followed by 10 μL of mixed ONI B-Cubed Imaging Buffer (Alfatest, Rome, Italy) for EV imaging. Two channels (2000 frames per channel) (647 and 488 nm) were acquired sequentially at 30 Hz in total internal reflection fluorescence (TIRF) mode. All images were analyzed using algorithms developed by ONI via their CODI website platform (https://alto.codi.bio/project/). Filtering and drift correction were performed using NimOS software following with temporal groups. The BD-Scan clustering tool was used to merge channels, with the bin size set to 20 and the maximum radius (r) set to 200 nm.

### Nano-flow cytometry (NanoFCM)

Nano-flow cytometry was performed using the Flow NanoAnalyzer (NanoFCM Inc.) to assess iMGEV size distribution and fluorescence-labeled EV populations. This system enables high-resolution measurement of fluorescence, particle size, and concentration, using quality control beads (S16M-Exo), size standards, and blanks according to the manufacturer’s protocol^68^. For EV labeling, samples were first diluted to 5 × 10^9^ particles/mL in dfPBS. Each staining reaction contained 2 μL of EV sample, 0.5 μL of pre-diluted (1:20) PSMA1-FITC antibody (Abbexa, Cat# abx317148), and 0.3 μL of 0.1% Triton X-100. The mixture was vortexed for 5 seconds and incubated overnight at 4°C in the dark. Following incubation, 1 μL of the labeled sample was further diluted 1:30 in dfPBS before analysis. Samples were then loaded into the NanoAnalyzer, and a boosting pressure was applied for 45 seconds prior to switching to sampling pressure. Data acquisition was performed over a 2-minute recording period. EV subpopulations were quantified using the instrument’s auto-threshold function.

### Protein extraction and concentration

iMG or iN cell lysates and iMGEVs were lysed in cold RIPA buffer (Cat# 89900, Thermo Fisher Scientific) supplemented with Halt protease and phosphatase inhibitor cocktail (Cat# 78442, Thermo Fisher Scientific) and sonicated for 2 mins. Protein concentration was determined by using Micro Bicinchoninic acid (BCA) assay (Cat# 23235, Thermo Fisher Scientific). All assays were incubated at 37°C for 120 min. The absorbance of the protein was read using a Biotek Synergy Mx plate reader at 562 nm.

### Data-independent-acquisition mass spectrometry (DIA-MS)

Equal amounts of protein (5 μg) from cell lysates and isolated iMGEVs were lysed in cold RIPA buffer supplemented with protease and phosphatase inhibitors and processed for data-independent-acquisition liquid chromatography-mass spectrometry analysis as previously described ^67^. Samples were flash-frozen on dry ice, then dried using a SpeedVac. Protein digestion was performed using the S-Trap Micro Spin protocol (PROTIFI, Cat# C02-micro).

Briefly, proteins were lysed in 10% SDS (Cat# L4509, SIGMA) with 100 mM triethylammonium bicarbonate (TEAB; Cat# 90114, Thermo Fisher Scientific), reduced with 10 mM dithiothreitol (DTT; Cat# 233153, SIGMA), and alkylated with 20 mM iodoacetamide (Cat# I6125, SIGMA). Following overnight trypsin (Cat# V511, Promega) digestion at 37°C, peptides were sequentially eluted using 40 μL each of 50 mM TEAB, 0.2% formic acid (FA; Cat# F6086, Aqua Solutions, Inc), and 50% acetonitrile (ACN; Cat# A955, Fisher Chemical), with each step spun at 4000 rpm for 1 minute. Eluates were pooled and dried via SpeedVac. Digested peptides were reconstituted in 20 μL of 0.1% FA in 5% ACN and centrifuged at 16,000 × g for 15 min. A total of 18 μL of the clarified supernatant was transferred to non-binding HPLC vials, and 4 μL was injected (1DDA + 3DIA) for analysis. Mass spectrometry was performed on a timsTOF Pro2 (Bruker) coupled to a nanoElute LC system (Bruker). Peptides were separated on an in-house-made 75 μm I.D. fused silica analytical column packed with 25-cm ReproSil-Pur C18-AQ (Dr. Maisch, GmbH, 120 Å, 3 μm) particles to a gravity-pulled tip at a flow rate of 500 nL/min. A 60-minute gradient was used for data-dependent acquisition (DDA)-PASEF and a 30-minute gradient for data-independent acquisition (DIA)-PASEF. The captive nano-electrospray voltage was set to 1600 V using a single-column configuration (no trap), with solvents consisting of Solvent A: 0.1% FA in water, and Solvent B: 0.1% FA in ACN.

#### DDA-PASEF Method:

Data-dependent acquisition using parallel accumulation-serial fragmentation (DDA-PASEF) was performed with 10 MS/MS PASEF scans per topN acquisition cycle. Ion accumulation and ramp times were set to 100 ms. The acquisition covered an m/z range of 100-1700 and an ion mobility range (1/K_0_) from 0.70 to 1.30 V·s/cm^2^. Collision energy was applied using a linear gradient based on ion mobility, ranging from 20 eV at 0.6 V·s/cm^2^ to 59 eV at 1.6 V·s/cm^2^, following default instrument parameters. Instrument calibration was performed using three reference ions (m/z 622, 922, and 1222) from the ESI-L Tuning Mix (Agilent).

Data independent acquisition-parallel accumulation serial fragmentation (DIA-PASEF) Method^69^: For data-independent acquisition (DIA-PASEF), samples were analyzed using 22 isolation windows, each 40 Da wide, spanning a mass range of 275-1155 Da. Each mass window was paired with a single ion mobility window covering 1/K_0_ from 0.70 to 1.30 V·s/cm^2^. The window scheme was optimized using the Window Editor tool in timsControl (Bruker) based on a representative DDA-PASEF run from the same sample set. Collision energy and calibration settings were identical to those used in the DDA-PASEF protocol.

### Proteomics analysis

The DIA-PASEF datasets were analyzed using the directDIA+ workflow in Spectronaut v18.2 (Biognosys), which enables library-free analysis without requiring a spectral library from DDA runs. Bruker raw data files were automatically converted to HTRMS format using the Spectronaut HTRMS Converter. Protein identification was performed by searching against a reviewed Human Swiss-Prot FASTA database (downloaded on 08/30/2024) using Pulsar, the integrated search engine in Spectronaut. Default factory settings were applied, including Trypsin/P as the enzyme, a peptide length range of 7-52 amino acids, carbamidomethylation of cysteines as a fixed modification, and oxidation (M) and N-terminal acetylation as variable modifications. False discovery rate (FDR) thresholds were set at 1% for peptide-spectrum matches (PSMs), peptides, and proteins. Protein quantification was performed at the MS2 level using the area under the extracted ion chromatogram (XIC), with automatic cross-run normalization and label-free quantification (LFQ).

Proteins identified in at least one of the three technical DIA injections were retained for downstream analysis. Comparative proteomic analysis between isogenic APOE3 and APOE4 iMGs and their derived EVs was performed using MetaboAnalyst v6.0^70^. Statistical analysis was conducted using a one-factor design (vehicle or fAβ treatment) with paired comparisons between APOE3 and APOE4 samples. Data were auto-scaled (mean-centered and divided by the standard deviation), and missing values were imputed using the limit of detection (LoD), defined as 1/5 of the minimum positive value for each variable. Principal component analysis (PCA), Pearson correlation, and volcano plots were proceeded and generated in MetaboAnalyst. For proteome-wide differential-abundance analyses, proteins meeting a nominal p value < 0.05 and an absolute fold-change > 1.2 but not the FDR threshold were considered exploratory candidates and were used for hypothesis generation and pathway-level analyses. Functional pathway analyses including Gene Ontology (GO) analysis and Kyoto Encyclopedia of Genes (KEGG) were performed using the DAVID online resources^71,72^ with false discovery rate (FDR) corrected *P* value <0.05. Circos plots, heatmaps, and pathway bubble plots were created using Sangerbox 3.0^73^. The bar plots were generated using Graphpad prism 10.

### Ingenuity pathway analysis (IPA)

We used the Ingenuity Pathway Analysis (IPA) (version 01-22, Qiagen) (https://www.qiagenbioinformatics.com/products/ingenuity-pathway-analysis/) to analyze, integrate, and categorize the proteomic data according to the software manual guidelines. A standard IPA core analysis was performed, including canonical pathways, upstream regulators, and disease and function annotation.

### Labeling of fAβ, fTau, and iMGEVs with PHrodo red dye

Fibrillar Aβ, fibrillar Tau (Tau-441 preformed fibrils; Cat# TF-1001-2, rPeptide), and iMGEVs were labeled with pHrodo red ester dye (Thermo Fisher Scientific, Cat# P36600) for cellular uptake assays. Prior to labeling, fAβ and fTau were sonicated using a Branson CPX2800H ultrasonic sonicator at the highest setting for 10 min to fragment the fibrils. For labeling, 20 μg of sonicated fAβ or fTau, or 20 μL of iMGEVs (at 5 × 10^9^ particles/mL), were incubated with 0.5 μL of pHrodo red dye (10.6 mM) at room temperature for 1 hour. To terminate the reaction and remove unbound dye, 350 μL of cold double-filtered PBS was added. Labeled samples were then concentrated and purified using Amicon Ultra-0.5 centrifugal filter columns (EMD Millipore): 10 kDa molecular weight cutoff (MWCO) for fAβ and fTau (Cat# UFC501096), and 100 kDa MWCO for EVs (Cat# UFC510096). Samples were centrifuged at 14,000 × g for 20 min. Final concentrations were adjusted to 1 μg/μL for labeled fAβ and fTau, and 1 × 10^9^ particles/mL for labeled iMGEVs for downstream uptake assays.

### fAβ and fTau uptake assays in iMGs

The uptake of fAβ and fTau by iMGs was assessed using live-cell imaging (IncuCyte S3, Sartorius), following a previously described method with minor modifications^60,74^. Briefly, isogenic APOE3 and APOE4 iMGs were seeded into 96-well plates (Corning, Cat# 3585) at a density of 15,000 cells per well and treated with a final concentration of 1 μg/mL of either pHrodo-labeled fAβ or fTau or fAβ+fTau. Time-lapse imaging was performed over a 48-hour period, capturing 9 fields per well every 30 min. Image data were analyzed using IncuCyte software, with the red fluorescent signal (from internalized pHrodo-labeled aggregates) used as a readout of phagocytic activity. Uptake efficiency was quantified as the total integrated red fluorescence intensity (OCU) per μm^2^ cell area at each time point. Four technical replicates were performed for each line.

### Uptake of labeled iMGEV by iPSC-derived neurons

iPSC-derived neurons were seeded into glass-bottom 96-well plates (Cellvis, Cat# P96-1.5H-N) at a density of 20,000 cells per well. On day 17 of differentiation (D17), neurons were treated with pHrodo-labeled iMGEVs at a dose of 2 × 10^6^ particles per well, corresponding to a particle-to-cell ratio of approximately 100:1. As a negative control, an equivalent volume of pHrodo dye (without EVs) was applied. Following a five-day incubation period, cells were fixed and subjected to immunostaining. Imaging was captured using a Leica SP8 confocal microscope. Red fluorescence intensity, indicating iMGEV uptake, was quantified using ImageJ by measuring the mean fluorescence intensity across the field of view.

### Confocal image processing and quantification

Images were acquired using a Leica SP8 confocal microscope in confocal mode with 10×, 20×, or 63× oil-immersion objectives, and a pinhole setting of 1.0 Airy unit. Confocal z-stacks were captured at 1024 × 1024 pixel resolution with system-optimized z-intervals. Post-acquisition, images were processed using Leica’s adaptive LIGHTNING deconvolution to enhance clarity and resolution. For imaging induced neurons, Ac-Caspase 3, AT8 and iMGEV uptake signals were captured at 1.5×, 4×, and 2× or 8× zoom using a 20× objective, respectively. In iMG/induced neuron co-culture experiments, imaging was performed at 2× zoom using the 63× oil objective. For whole organoid imaging, Tile Scan mode was employed, and images were stitched using Leica software. Quantification of Ac-Caspase 3, AT8, and iMGEV uptake was performed using ImageJ (Fiji). Colocalization analyses in iMG/induced neuron co-cultures and NF-κB signal analysis in organoids were conducted using Imaris software (Bitplane). The Imaris surface function was used with auto-thresholding to ensure objective quantification. Total and nuclear NF-κB signals were separately assessed by masking and surface rendering within IBA1^+^ cells and nuclei, respectively.

### Calcium imaging of induced neurons by IncuCyte S3

WT iNs were seeded into 96-well plates at a density of 20,000 cells per well and transduced on day 9 (D9) with 2 μL/well of NeuroBurst Orange Lentivirus (Sartorius, Cat# 4736), which expresses a genetically encoded calcium indicator (cGAMP6) with an orange fluorescence under the promoter of human SYNAPSIN1 (hSYN1), in 100 μL of culture medium. After a 24-h incubation, the virus-containing medium was replaced with fresh iN maintenance medium. Neurons were then cultured for an additional 7 days prior to EV treatment. On day 17, unlabeled iMGEVs were added at a dose of 2 × 10^6^ particles per well. Live-cell calcium imaging was conducted using the IncuCyte S3 system (Sartorius), capturing fluorescent images every 12 h over a 5-day period. This allowed for real-time monitoring of the temporal dynamics of neuronal calcium signaling in response to EV exposure. Calcium activity was quantified using the IncuCyte S3 neuronal activity analysis software, focusing on key metrics including neuronal burst strength (OCU), burst rate (events/minute), burst duration (seconds), and synchrony (mean correlation). Three independent experiments were conducted for the calcium imaging analysis.

### Neuronal activity analyses with HD-MEA

We used CMOS-(HD)-MEA platform (MaxWell Biosystems AG, Switzerland) for recording iPSC-neuron activity upon iMGEV treatment. To prepare the plates for neuron plating, MaxOne^+^ chips were pre-conditioned with sterile neuronal culturing media for 2 days at 37°C, followed by coated with poly-L-ornithine (10 μg/mL) and mouse laminin 20 μg/ml at 37°C for 1 day. Chips were then washed with deionized sterile water for three times and air-dried for 1 h at room temperature. NGN2-induced WT iNs on day 4 of differentiation were plated on the single well of HD-MEA plate and recorded similarly as described^75^. For plating, 500,000 neurons in a 50 μL droplet were plated on top of the electrode array center in each chip. The chip was covered with a sterile MaxOne lid and incubated in a 5% CO_2_ incubator at 37°C, with RH > 95% for 1 hour. After incubation, each chip was carefully filled with 0.6 mL of pre-warmed complete culture medium. On day 25, baseline activity scans of iNs were acquired on the HD-MEA platform to record spontaneous spiking. fAβ-treated *APOE3* or *APOE4* iMGEVs were then applied at 5 × 10^7^ particles per chip, and full activity scans were recorded longitudinally for three additional days. Prior to each recording, the HD-MEA system was equilibrated to 37 °C and 5% CO_2_. Data were exported for downstream processing and statistical analysis using GraphPad Prism.

### Proteasomal activity assay

The chymotrypsin-like, trypsin-like, and caspase-like activities of constitutive proteasomes were assessed using the Proteasome Activity Fluorometric Assay Kit (UBPbio, Cat# J4120), following the manufacturer’s instructions. Briefly, WT iNs were seeded into 96-well plates at a density of 20,000 cells per well. On day 17 of differentiation (D17), neurons were treated with either vehicle (PBS) or iMGEVs at a dose of 2 × 10^6^ particles per well, corresponding to a particle-to-cell ratio of 100:1. After a 7-day incubation, cells were lysed in 20 μL of ice-cold lysis buffer (40 mM Tris, pH 7.2, 50 mM NaCl, 2 mM β-mercaptoethanol, 2 mM ATP, 5 mM MgCl_2_, and 10% glycerol) and sonicated for 1 minute. Lysates were then centrifuged at 17,000 × g for 20 min at 4°C to remove debris. Fluorogenic substrates Suc-LLVY-AMC, Boc-LRR-AMC, and Z-LLE-AMC were used to measure chymotrypsin-like, trypsin-like, and caspase-like proteasomal activities, respectively. Substrate cleavage by proteasomes released the AMC fluorophore, and fluorescence intensity was recorded in kinetic mode for 14 min using a BioTek Synergy II plate reader (excitation/emission: 360/460 nm). Proteasome activity was quantified by calculating the slope of fluorescence increase over time using the formula (Y_14_ – Y_0_)/14, where Y_14_ and Y_0_ represent fluorescence values at 14 and 0 min, respectively. Slope values for each substrate were compared across treatment groups to assess changes in proteasome activity. Three independent experiments were conducted for each assay.

### Western Blot

Equivalent amounts (5 μg) of iMG cell lysates from three pairs of isogenic lines or MAPT P301L iN cell lysates were measured using the microBCA kit and denatured in Laemmli sample buffer (Cat# 1610737, Bio-Rad). For assessment of 3R and 4R tau isoform expression, iN lysates were treated with lambda protein phosphatase (Cat# P0753S, New England Biolabs) at 30°C for 60 min, according to the manufacturer’s instructions, to dephosphorylate tau before sample denaturation. A recombinant tau protein ladder (Cat# T7951, MilliporeSigma) was included to confirm the migration and identity of the 3R and 4R tau isoforms. The protein samples were then loaded onto 4-12% SDS-PAGE gels (Cat# 4561096, Bio-Rad) and electro-transferred to 0.45 μm nitrocellulose membranes (Cat# 1620115, Bio-Rad). The membranes were blocked with 5% non-fat milk (Cat# 9999S, Cell Signaling Technology) for 1 hour and incubated overnight at 4°C with primary antibodies. The following primary antibodies were used for western blot: anti- NF-κB p65 (1:100, Cat# sc-372, Santa Cruz), anti-phospho NF-κB p65 (1:1000, Cat# 3033S, Cell Signaling Technology), anti-3R Tau (1:1000, Cat# 05-803, Millipore), anti-4R Tau (1:1000, Cat# 823701, BioLegend), anti-phospho-Tau (Ser202, Thr205) (AT8; 1:1000, Cat# MN1020B, Invitrogen), anti-total Tau (1:2000, Cat# A0024, Dako) and anti-beta actin (1:1000, Cat# sc-130065, Santa Cruz). The membranes were then incubated with horseradish peroxidase (HRP)-labeled secondary antibodies (anti-mouse IgG, HRP-linked antibody, Cat# 7076S, Cell Signaling Technology) for 1 h. Immunoreactivity was detected using enhanced chemiluminescence solutions (Millipore, Cat# WBKLS0100). For the quantification analysis, a rectangular area was selected enclosing the bands of interest, and the intensity of each band was measured from the area showing the most intense signal using ImageJ. The signal intensities of all protein bands were normalized by the β-actin band or the total protein staining by using Swift Membrane Stain (Cat# 786-677, G Biosciences) as indicated.

### Enzyme-linked immunosorbent assay (ELISA)

P301L Tau induced neurons were seeded into 96-well plates at a density of 20,000 cells per well. On day 17 of differentiation (D17), neurons were treated with vehicle (PBS) or iMGEVs at a dose of 2 × 10^6^ particles per well, corresponding to a particle-to-cell ratio of approximately 100:1. Following a seven-day incubation period, cells were lysed in RIPA buffer supplemented with protease and phosphatase inhibitor cocktail and 1% Triton X-100. Commercial ELISA kits were used to evaluate the protein concentration of total NF-κB p65 level in iMG cell lysates (Cat# 85-86083-11, Thermo Fisher Scientific), total Tau (Cat# KHB0041, Thermo Fisher Scientific), pThr181 Tau (Cat# KHO0631, Thermo Fisher Scientific) and pSer396 (Cat# KHB7031, Thermo Fisher Scientific) in neuronal cell lysates according to the manufacturer’s instructions. Duplicated wells were performed in each assay. Two independent experiments were conducted for each assay.

### Statistical analysis

Data are presented as the mean ± standard error of the mean (SEM) unless otherwise indicated. Statistical analyses were performed using GraphPad Prism (version 10.0). Distribution of the raw data was tested for normality of distribution. Statistical analyses were performed using Wilcoxon-Mann-Whitney test, Receiver Operating Characteristic (ROC) analysis or ANOVA followed by multiple comparison tests as indicated in the figure legends. Statistical significance was defined as p value <0.05. P values in functional enrichment analyses were adjusted for multiple comparisons using FDR correction with the Benjamini-Hochberg method where indicated.

## Supplementary Material

This is a list of supplementary files associated with this preprint. Click to download.

- revisedSupplementarytables.xlsx
- SupplementaryInformationR1notrack.pdf
- r.pdf

## Figures and Tables

**Figure 1.**
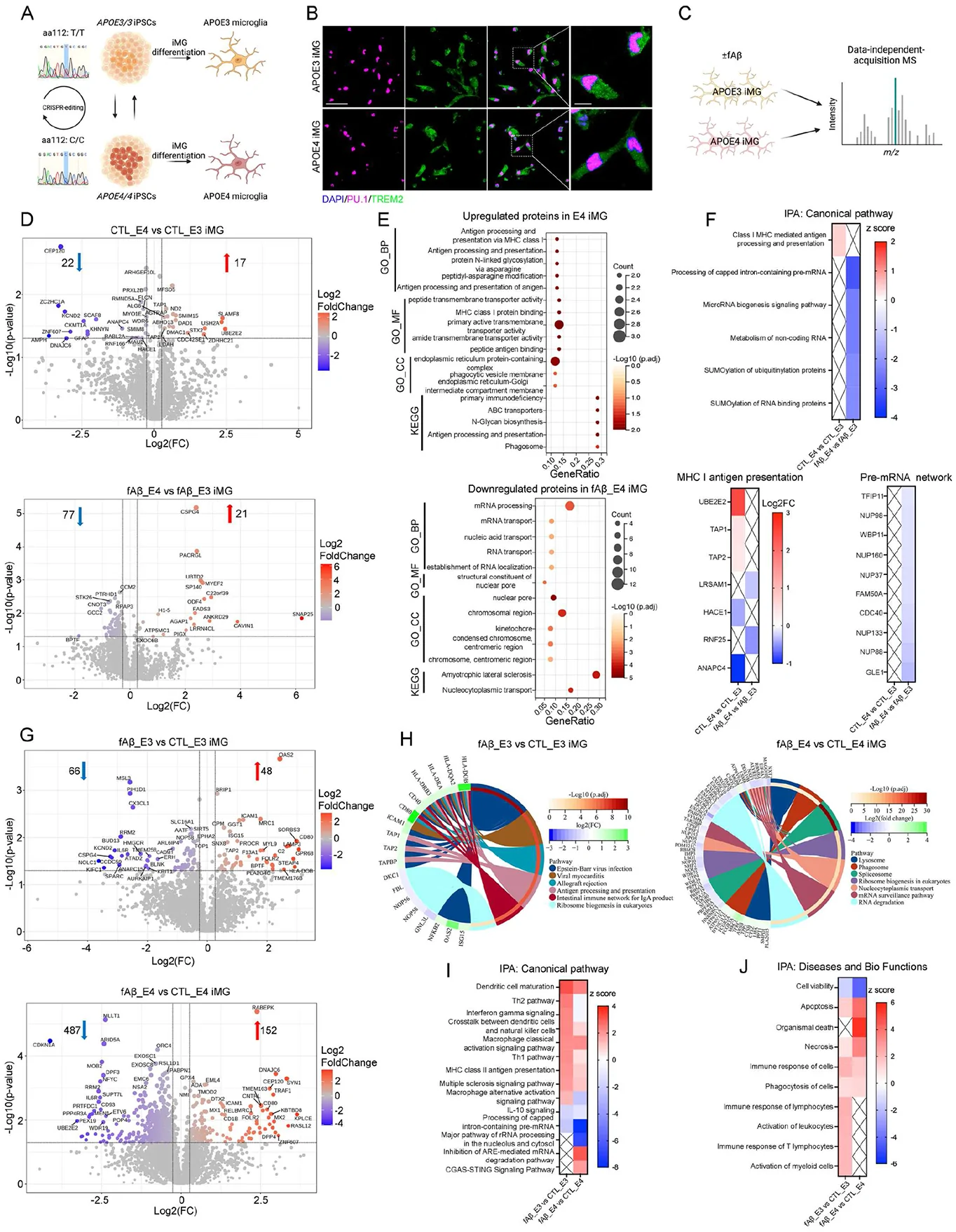
Isogenic *APOE3* and *APOE4* iPSC-derived microglia exhibit distinct proteomic signatures under basal and fibrillar amyloid β (fAβ) stimulated conditions. **A,** Schematic illustrating the generation of three pairs of isogenic human iPSC lines homozygous for *APOE3* or *APOE4* alleles using CRISPR-Cas9 editing, followed by differentiation into microglia-like cells (iMGs). **B,** Representative immunostaining images confirming the expression of microglial markers PU.1 and TREM2 in *APOE3* and *APOE4* iMGs. **C,** Experimental workflow for quantitative proteomic profiling of *APOE3* and *APOE4* iMGs under basal and fAβ-treated conditions using DIA-MS. **D,** Volcano plots showing the significantly differentially expressed proteins (sDEPs) between *APOE4* and *APOE3* iMGs under basal conditions (top) and following Aβ stimulation (bottom). Significantly regulated proteins with the threshold of p < 0.05 and ∣fold change∣ > 1.2 are highlighted in red (upregulation) and blue (downregulation) color. **E,** Gene Ontology (GO) and KEGG pathway analyses identifying enriched pathways in proteins significantly altered between *APOE4* and *APOE3* iMGs at baseline (upregulated proteins) and following fAβ exposure (downregulated proteins). **F,** Ingenuity pathway analysis (IPA) showing activation of MHC class I-mediated antigen presentation pathways in basal *APOE4* iMGs and suppression of RNA processing and SUMOylation of RNA-binding proteins following Aβ exposure, relative to *APOE3* iMGs. **G,** Volcano plots depicting within-genotype proteomic differences by comparing fAβ-treated to baseline conditions in *APOE3* (top) and *APOE4* (bottom) iMGs. **H,** Circos plots highlighting the significantly altered proteins by comparing within-*APOE* genotype responses to fAβ stimulation. Green color indicates upregulated proteins in fAβ-treated iMGs compared to basal iMGs, while blue color indicates downregulated proteins. **I,** IPA analysis comparing *APOE* genotype-specific canonical pathway changes following fAβ treatment versus basal conditions. **J,** IPA-based predictions of biological functions, revealing enhanced immune activation in *APOE3* iMGs and elevated apoptosis and cell death signatures in *APOE4* iMGs following fAβ stimulation.

**Figure 2.**
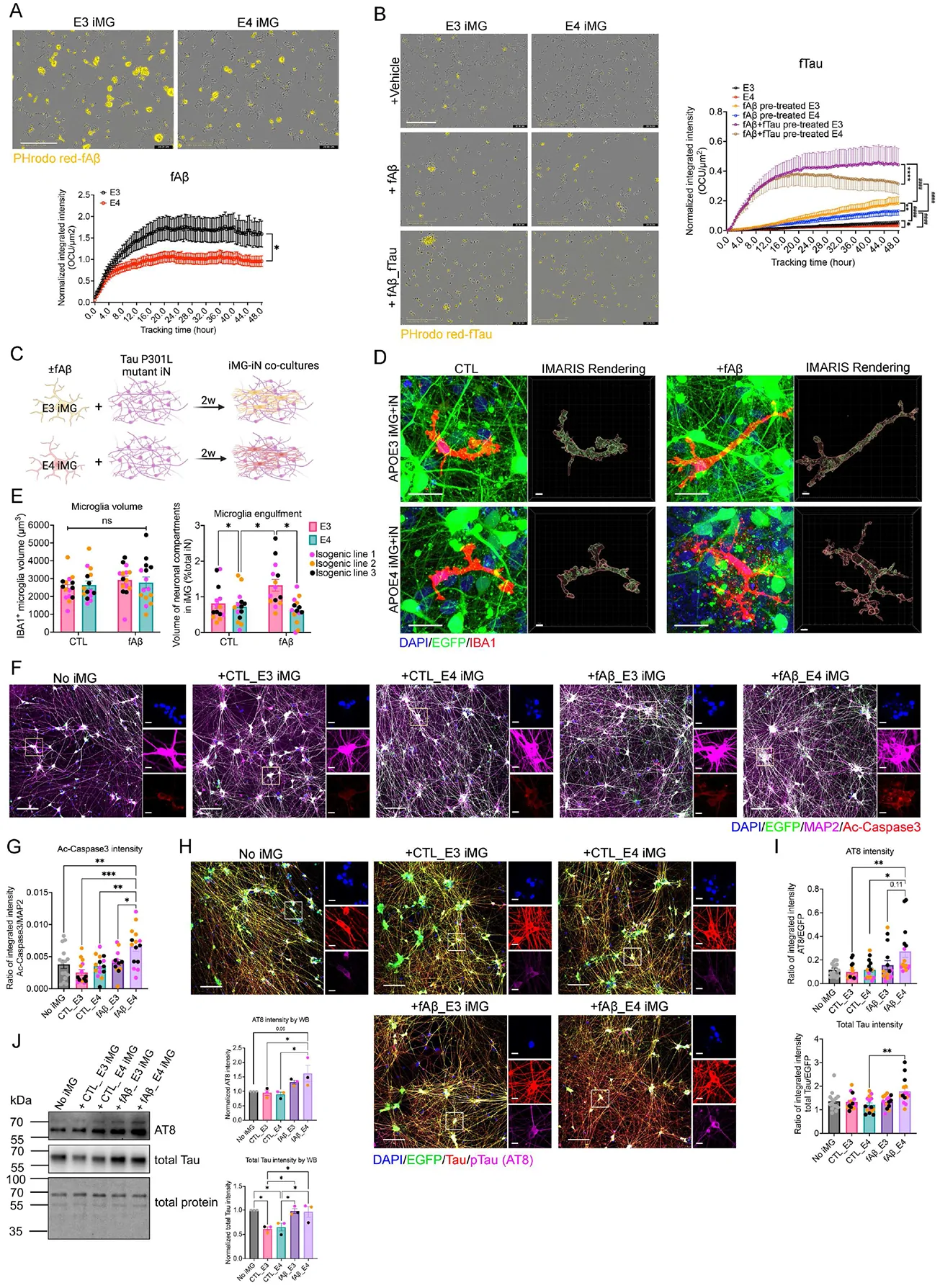
*APOE4* microglia exhibit impaired phagocytic function and exacerbate neuronal damage in a human iPSC-derived microglia-neuron co-culture system. **A,** Live-cell imaging of pHrodo-labeled fibrillar Aβ uptake by *APOE3* and *APOE4* iMGs over 2 days by IncuCyte. N = 11 from three pairs of isogenic lines (3-4 repeats per line). Data shown as mean ± SEM. *p < 0.05. Two-way ANOVA. Scale bar, 200 μm. **B,** Live-cell imaging of pHrodo-labeled fTau by *APOE3* and *APOE4* iMGs following pretreatment with vehicle, fAβ alone, or combined fAβ and fTau. Cells were pre-treated with vehicle or fAβ alone, or fAβ/fTau for three days. N = 11~12 from three pairs of isogenic lines (3 to 4 repeats per line). Data shown as mean ± SEM. *p < 0.05, **p < 0.01, ****p < 0.0001, ^####^p<0.0001. Receiver Operating Characteristic (ROC) analysis was used. Scale bar, 200 μm. **C,** Experimental schematic showing the co-culture of *APOE3* or *APOE4* iMGs with iPSC-derived neurons carrying the P301L Tau mutation. iMGs were pre-treated with vehicle or fAβ, followed by a two-week co-culture period. **D,** Representative confocal images and IMARIS 3D-rendered reconstructions illustrating the morphology of *APOE3* and *APOE4* iMGs (IBA1^+^, red) and their engulfment of EGFP-expressing neurons (green) under basal (CTL) or fAβ-treated conditions. Scale bar, 25 μm and 5 μm. **E,** Quantification of microglial volume and co-localized volume indicative of microglia-neuron interactions. N > 14 single microglia from iMG/iN coculture with three pairs of isogenic lines were included for each group. Data were presented as mean ± SEM. *p < 0.05. ns, not significant. Two-way ANOVA followed by Tukey’s multiple comparisons test. **F,** Representative immunofluorescence images illustrating neuronal apoptosis (activated caspase-3, red) in P301L Tau neurons (MAP2, Magenta) cultured alone or co-cultured with *APOE3* or *APOE4* iMGs under basal or fAβ-treated conditions. Scale bar, 100 μm and 10 μm. **G,** Quantification of neuronal apoptosis measured by activated caspase-3 intensity normalized to MAP2 intensity. N = 15 images from iMG/iN co-culture with three pairs of isogenic lines were quantified for each group. Data shown as mean ± SEM. *p < 0.05, **p < 0.01, ***p < 0.001. Ordinary one-way ANOVA with Tukey’s multiple comparisons test. **H,** Representative immunofluorescence images showing pathological tau phosphorylation (AT8, magenta) and total Tau (red) in EGFP-expressing neurons (green) cultured alone or co-cultured with *APOE3* or *APOE4* iMGs under basal or fAβ-treated conditions. N = 15 images from iMG/iN co-culture with three pairs of isogenic lines were quantified for each group. Scale bar, 100 μm and 10 μm. **I,** Quantification of tau phosphorylation (AT8 intensity normalized to EGFP) and total Tau signals (total Tau normalized to EGFP). Data presented as mean ± SEM. *p < 0.05, **p < 0.01, Kruskal-Wallis test with Dunn’s multiple comparisons test. **J,** Immunoblotting and quantification of phosphorylated tau (AT8) and total tau in P301L iNs cultured alone (No iMG) or co-cultured with *APOE3* or *APOE4* iMGs under basal conditions or following fAβ stimulation of the iMGs. AT8 and total tau intensity was normalized to total protein staining, and relative protein levels to No iMG group were calculated. Each point represents an individual isogenic line. Data represent mean ± SEM. *p < 0.05. RM one-way ANOVA with Tukey’s multiple comparisons test.

**Figure 3.**
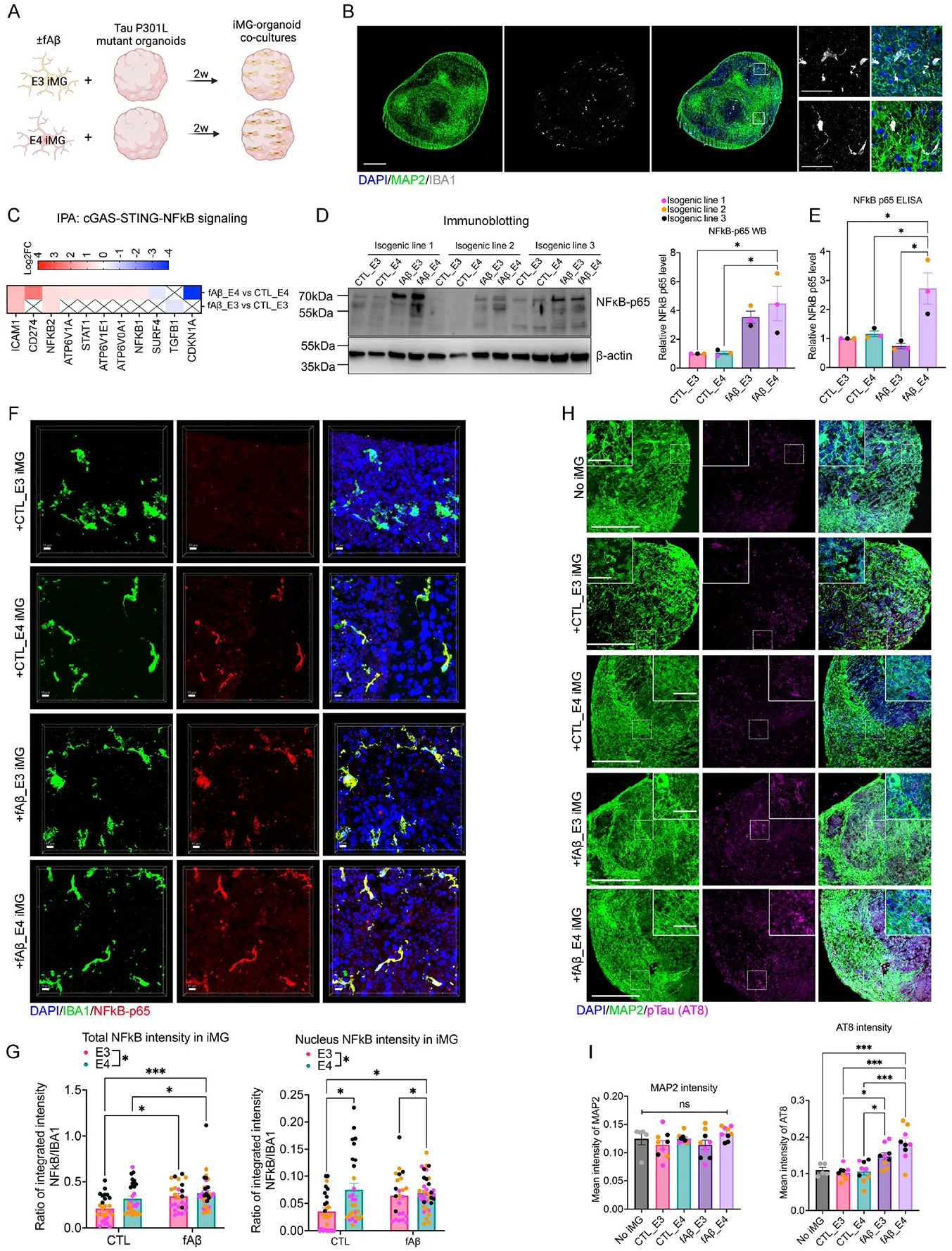
*APOE4* iMGs enhance NF-κB activation and tau pathology in a 3D iMG-brain organoid co-culture model. **A,** Schematic overview of the experimental design. Basal and fAβ-treated isogenic *APOE3* or *APOE4* iMGs were added to 6-month-old P301L Tau mutant brain organoids, followed by a 2-week co-culture period. **B,** Confocal image showing integration of IBA1^+^ iMGs with MAP2^+^ neurons within the organoids. Scale bar, 250 μm and 50 μm. **C,** Ingenuity Pathway Analysis of APOE4 versus *APOE3* iMGs showing increased activation of key proteins involved in the cGAS-STING-NF-κB signaling pathway following fAβ stimulation. **D,** Immunoblotting and quantification of NF-κB p65 in iMGs from three independent *APOE* isogenic pairs under CTL or fAβ-treated conditions. NF-κB p65 level was normalized to β-actin, and relative protein levels to CTL_E3 group were calculated. Each point represents an individual isogenic line. Data represent mean ± SEM. *p < 0.05. RM one-way ANOVA with Tukey’s multiple comparisons test. **E,** ELISA quantification of NF-κB p65 in iMG lysates under CTL or fAβ-treated conditions from three independent *APOE* isogenic pairs. Relative NF-κB p65 levels to CTL_E3 group were calculated. Data represent mean ± SEM. *p < 0.05, **p < 0.01. RM one-way ANOVA with Tukey’s multiple comparisons test. **F,** Representative images of NF-κB signal in co-cultured organoids. IBA1 (green), NF-κB p65 (red), and DAPI (blue) staining show increased NF-κB expression in *APOE4* iMGs under fAβ conditions. Scale bar, 10 μm. **G,** Quantification of total and nuclear NF-κB intensity in *APOE3* and *APOE4* iMGs under basal and fAβ-treated conditions. At least 3 images/organoid from 3 iMG-organoid co-cultures for each line were quantified. Each point represents an individual image. Data represent mean ± SEM. *p < 0.05, ***p < 0.001. Two-way ANOVA with Tukey’s multiple comparisons. ANOVA p value was assessed to compare *APOE* genotype effect. **H,** Representative images of AT8 staining in iMG-organoids co-cultures among different groups. Increased AT8 signal is observed in organoids treated with *APOE4* iMGs, especially under fAβ stimulation. Scale bar, 250 μm and 50 μm. **I,** Quantification of AT8 and MAP2 intensities in organoids. *APOE4* iMGs significantly elevated tau phosphorylation in P301L Tau mutant organoids, while MAP2 levels remained unchanged. N = 5~9 organoids/group. At least 3 iMG-organoid co-cultures for each line were used. Each point represents an individual image. Data are shown as mean ± SEM. *p < 0.05, ***p < 0.001. Ordinary one-way ANOVA with Tukey’s multiple comparisons test.

**Figure 4.**
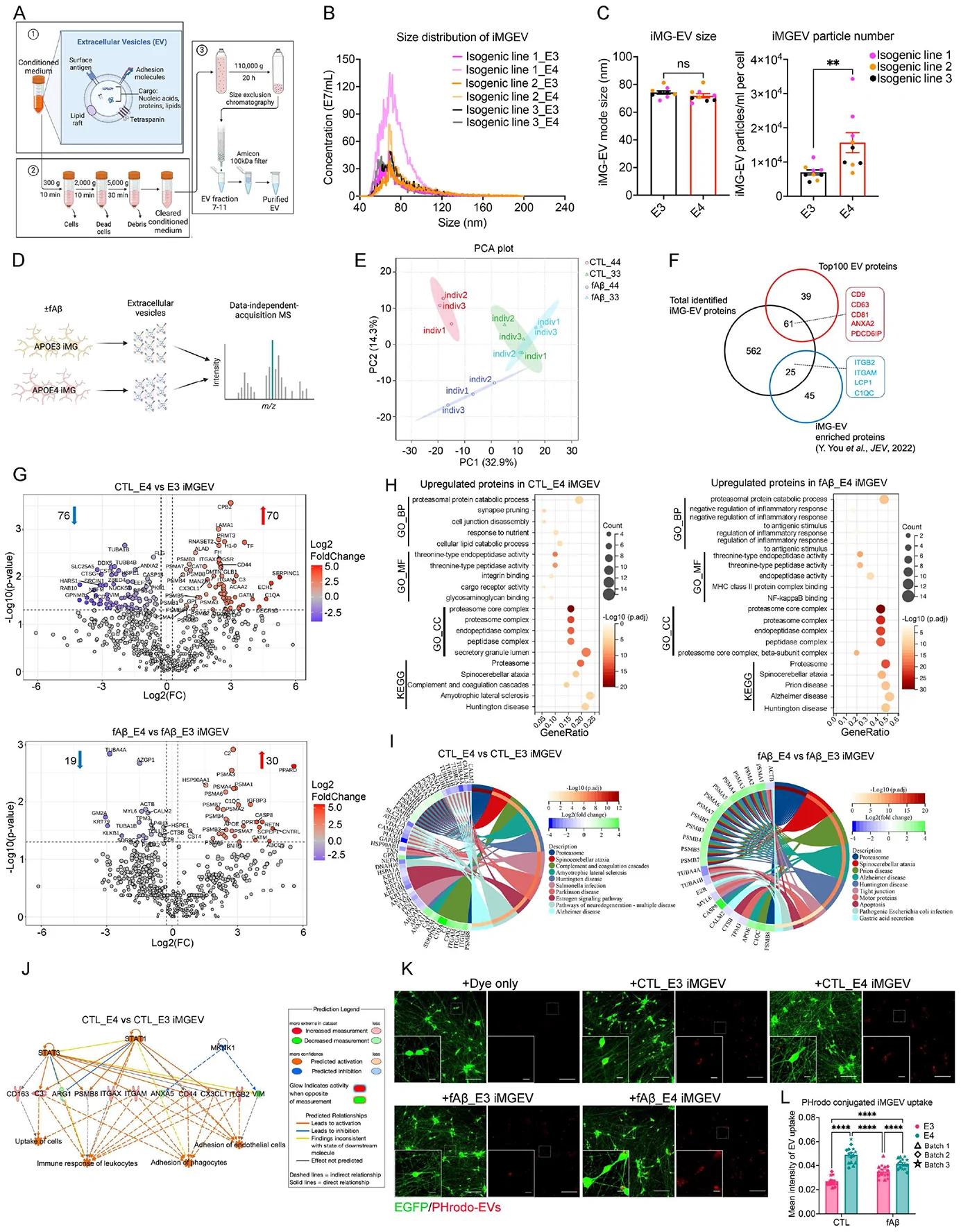
Proteomic analysis of extracellular vesicles from *APOE3* and *APOE4* iPSC-derived microglia under basal and fAβ-stimulated conditions. **A,** Schematic workflow illustrating isolation and purification of EVs from conditioned medium of *APOE3* and *APOE4* iMG cultures using sequential centrifugation and size exclusion chromatography. **B,** Size distribution of isolated iMGEVs from three independent isogenic *APOE3* and *APOE4* iMG lines, measured by nano-flow cytometry (NanoFCM). **C,** Quantification of iMGEV mode size and particle number per cell in different APOE genotypes. N = 9 in each group (3 repeats/line × 3 isogenic lines). Data presented as mean ± SEM. ns, not significant. **p < 0.01. Mann-Whiteney test was applied. **D,** Experimental design for quantitative proteomic profiling of EVs derived from *APOE3* and *APOE4* iMGs under basal and fAβ-stimulated conditions using DIA-MS. **E,** Principal component analysis (PCA) demonstrating distinct proteomic clustering of iMGEVs according to genotype and treatment. **F,** Venn diagram showing identified proteins in iMGEVs overlapping with top 100 small EV proteins (http://www.exocarta.org/) and the published microglial EV enriched proteins ^36^. **G,** Volcano plots showing significantly differentially expressed EV proteins between APOE4 and *APOE3* iMGEVs under basal conditions (top panel) and following fAβ stimulation (bottom panel). Significantly regulated proteins with the threshold of p < 0.05 and ∣fold change∣ > 1.2 are highlighted in red (upregulation) and blue (downregulation) color. **H,** GO and KEGG pathway enrichment analyses of proteins significantly upregulated in *APOE4* iMGEVs relative to *APOE3* under basal (left) and fAβ-treated conditions (right). **I,** Circos plots highlighting the significantly altered proteins by comparing *APOE4* and *APOE3* iMGEVs at baseline (left) and following fAβ stimulation (right). Green color indicates upregulated proteins in *APOE4* iMGEVs compared to *APOE3* iMGEVs, while blue color indicates downregulated proteins. **J,** IPA network analysis highlighting key upstream regulators and downstream biological processes predicted from EV protein alterations in *APOE4* compared to *APOE3* at baseline. **K,** Representative images illustrating uptake of pHrodo red dye-labeled EVs (pHrodo-EVs, red) by EGFP-expressing neurons (green). Scale bar, 100 μm and 10 μm. **L,** Quantification of neuronal EV uptake demonstrating significantly higher internalization of *APOE4* iMGEVs compared to *APOE3* iMGEVs, especially under Aβ conditions. N = 15 images from three independent experiments were quantified for each group. Each point represents an individual image, with symbols indicating three independent experimental batches. Data were shown as mean ± SEM. ****p < 0.0001. Two-way ANOVA with Tukey’s multiple comparisons test.

**Figure 5.**
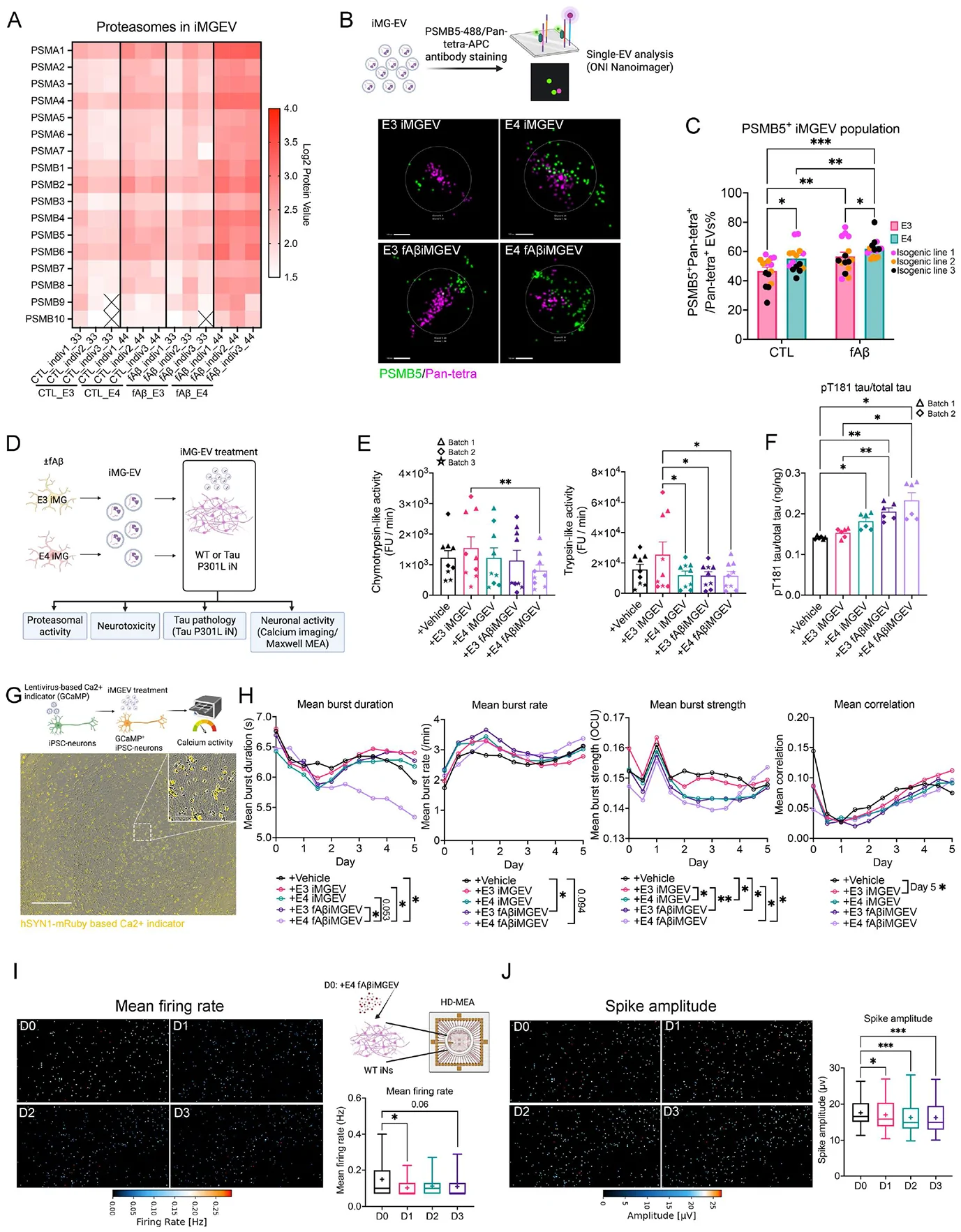
*APOE4* iMGEVs impair neuronal proteasome activity, promote tau pathology and dampen neuronal activity. **A,** Heatmap showing expression levels of 20S proteasome subunits in iMGEVs across *APOE3* and *APOE4* genotypes under basal and fAβ-treated conditions. B, Single-EV multiplex imaging using PSMB5-488 and pan-tetraspanin (Pan-tetra)-APC antibody labeling on an ONI Nanoimager. Representative images of E3 and E4 iMGEVs under CTL or fAβ-treated conditions showing PSMB5 (green) and Pan-tetra (magenta) at a single-EV level. Scale bars, 100μm. **C,** Quantification of PSMB5^+^ EVs population in *APOE3* and *APOE4* iMGEVs under basal and fAβ-treated conditions by super-resolution microscopy. Each dot represents an independent image. N = 5 images per line from three pairs of isogenic lines. Data were shown as mean ± SEM. *p < 0.05, **p < 0.01, ***p < 0.001. Two-way ANOVA with Tukey’s multiple comparisons test. **D,** Schematic overview of experimental design for evaluating iMGEV effects on neuron functions. **E,** Chymotrypsin-like and trypsin-like proteasomal activities in neurons treated with basal and fAβ-stimulated *APOE3* and *APOE4* iMGEVs for 7 days. FU, fluorescence unit. N = 9 per group. Shapes indicate three independent experimental batches. Individual points represent technical replicate wells from the indicated batch. Data were shown as mean ± SEM. *p < 0.05. RM one-way ANOVA with Holm-Sidak’s multiple comparisons test. **F,** pTau181 ELISA of P301L Tau induced neurons following 7-day iMGEV treatment. N = 6 per group. Shapes indicate two independent experimental batches. Individual points represent technical replicate wells from the indicated batch. Data were shown as mean ± SEM. *p < 0.05, **p < 0.01. Brown-Forsythe and Welch ANOVA with Dunnett’s T3 multiple comparisons test. **G,** Representative image of lentivirus infected neurons expressing hSYN1-mRuby-GCaMP6 used for real-time Ca^2+^ imaging. Scale bar, 100 μm and 25 μm. **H,** Time-course analysis of neuronal activities (mean burst duration, rate, strength and correlation) after iMGEVs treatment using IncuCyte S3 live-cell imaging over 5 days. N = 4-5 replicates per batch from three independent batches. Data were shown as mean value. *p < 0.05, **p < 0.01. Receiver Operating Characteristic (ROC) analysis was used for mean burst duration, rate and strength. Two-way ANOVA with Tukey’s multiple comparisons test was used for mean correlation. **I,** High-density multielectrode array (HD-MEA) recordings of mean firing rate in D25 WT iNs following exposure to fAβ-treated E4iMGEVs at D0 with longitudinal followup for 3 days (D1-D3). Data were shown with the Box and whiskers plot (Min to Max). The cross line indicates mean value. *p < 0.05, ***p < 0.001. Kruskal-Wallis test with Dunn’s multiple comparisons test was used. **J,** Quantification of spike amplitude in D25 WT iNs following exposure to fAβ-treated E4iMGEVs at D0 with longitudinal follow-up for 3 days (D1-D3). Data were shown with the Box and whiskers plot (Min to Max). *p < 0.05, ***p < 0.001. Kruskal-Wallis test with Dunn’s multiple comparisons test was used.

**Figure 6.**
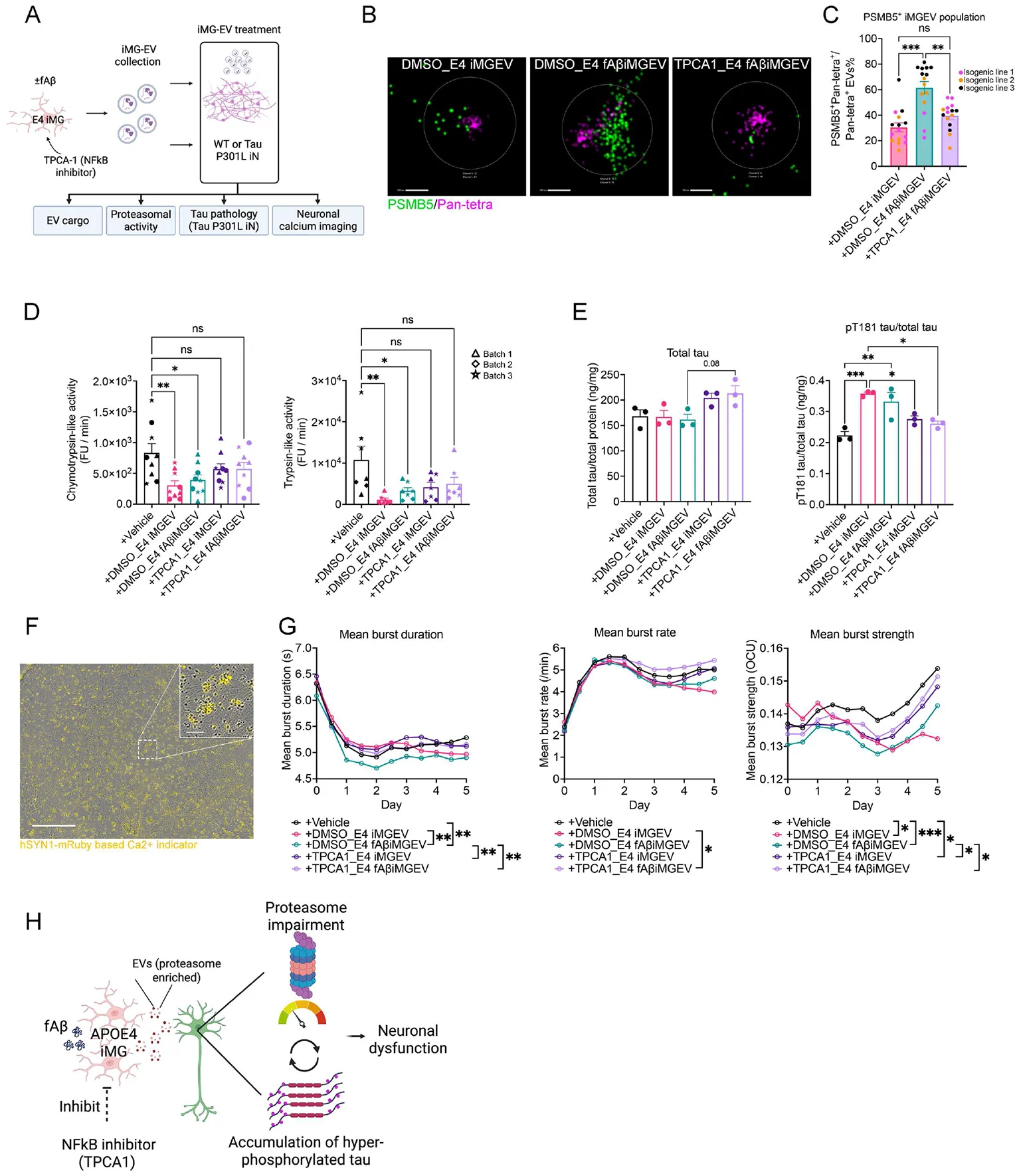
NF-κB inhibition in *APOE4* microglia under basal and fAβ-stimulated conditions attenuates proteasome-enriched EV cargo and partially rescues iMGEV-driven neuronal dysfunction. **A,** Experimental design. *APOE4* iMGs were pretreated with vehicle (DMSO) or the NF-κB inhibitor TPCA1 (0.1μM) for 2 hours, followed by exposure to control or fAβ conditions. Microglial EVs were then isolated and applied to WT or P301L Tau iNs for downstream analyses. **B,** Single-EV multiplex imaging on an ONI Nanoimager using PSMB5-488 (green) and pantetraspanin (Pan-tetra)-APC (magenta) antibody labeling. Representative images of DMSO_E4 iMGEV, DMSO_E4 fAβ iMGEV, and TPCA1_E4 fAβ iMGEV showing PSMB5 and Pan-tetra at single-EV resolution. Scale bars, 100 μm. **C,** Quantification of the PSMB5^+^ EV population (PSMB5^+^Pan-tetra^+^/Pan-tetra^+^ EVs%) across different groups. Each dot represents an independent image. Images were collected from three pairs of isogenic lines. Data are shown as mean ± SEM. **p < 0.01, ***p < 0.001, ns, no significance. Brown-Forsythe and Welch ANOVA with Dunnett’s T3 multiple comparisons test. **D,** Neuronal proteasome activity assays following 7-day treatment with indicated *APOE4* iMGEV preparations (vehicle, DMSO_E4 iMGEV, DMSO_E4 fAβ iMGEV, TPCA1_E4 iMGEV, TPCA1_E4 fAβ iMGEV). FU, fluorescence unit. N = 7-9 per group. Shapes indicate three independent experimental batches. Individual points represent technical replicate wells from the indicated batch. Data were shown as mean ± SEM. *p < 0.05, **p < 0.01, ns, no significance. RM one-way ANOVA with Holm-Sidak’s multiple comparisons test. **E,** Tau ELISA in P301L Tau iNs after 7-day EV exposure, showing total tau and pT181 tau/total tau ratio across indicated groups. N = 3 per group. Data were shown as mean ± SEM. *p < 0.05, **p < 0.01, ***p < 0.001. One-way ANOVA with Tukey’s multiple comparisons test. **F,** Representative image of lentivirus infected neurons expressing hSYN1-mRuby-GCaMP6 used for real-time Ca^2+^ imaging. Scale bar, 100 μm and 25 μm. **G,** Time-course analysis of neuronal network activity metrics (mean burst duration, burst rate, and burst strength) over 5 days following treatment with *APOE4* iMGEVs generated under DMSO or TPCA1 conditions. N = 17 to 23 per group from three independent experiments. Data were shown as mean value. *p < 0.05, **p < 0.01, ***p < 0.001. Receiver Operating Characteristic (ROC) analysis was used. **H,** Schematic figure illustrating the potential mechanisms by which *APOE4* iMGEVs, particularly under fAβ challenge, contribute to disease progression in neurodegeneration and how targeting NF-κB signaling may provide therapeutic leverage. Under fAβ challenge, *APOE4* iMGs produce proteasome-enriched EVs that impair neuronal proteostasis, promote tau phosphorylation, and disrupt neuronal activity. NF-κB inhibition (TPCA1) reduces proteasome-enriched EV cargo and partially mitigates these downstream neuronal effects.

## Data Availability

The data presented in this study is available in this article and supplementary material. The raw data files from mass spectrometry were deposited to the ProteomeXchange Consortium via the PRIDE partner repository with the dataset identifier PXD074177 and are available at the following URL: http://www.ebi.ac.uk/pride. Source Data are provided with this paper.
